# Competitive Meso-Damage Model Dependent on Stress State for Advanced High-Strength Steels

**DOI:** 10.3390/ma19163390

**Published:** 2026-08-10

**Authors:** Hongpai Zhu, Di Li, Junjie Liu, Jinbing Ding, Wancong Xu

**Affiliations:** School of Transportation and Vehicle Engineering, Shandong University of Technology, Zibo 255000, China; 18179393107@163.com (H.Z.); 13256390277@163.com (J.L.); 13345026939@163.com (J.D.); 19163546355@163.com (W.X.)

**Keywords:** advanced high-strength steel, competitive fracture, microdamage mechanics, GTN, stress triaxiality, Lode angle

## Abstract

Advanced High-Strength Steel (AHSS) exhibits stress-state-dependent competing shear–tensile fracture modes that limit the applicability of conventional ductile fracture criteria based solely on equivalent plastic strain accumulation, such as the Forming Limit Diagram (FLD) approach and the classical Gurson–Tvergaard–Needleman (GTN) model. This paper proposes an extended GTN damage model incorporating Hill’48 anisotropy and the Nahshon–Hutchinson shear mechanism, regulated by a stress-state-dependent weighting function. The experimental program comprised uniaxial tension tests for constitutive calibration, notched plate specimens with shear angles ranging from 0° to 90° (spanning pure shear to tensile–shear stress states), and tension-bending tests. The fracture initiation point was identified from the abrupt load drop on the experimental force–displacement curve and further located in the finite element simulation to extract the corresponding stress-state history. SEM fractography was employed to characterize the microscopic damage mechanisms, revealing a continuous transition from shear-dominated to void-dominated damage at a critical stress triaxiality of approximately 0.35. A weighting function dependent on both stress triaxiality and the normalized Lode angle was formulated to couple void evolution with shear band localization. Following calibration via finite element inverse fitting, the model, implemented as an ABAQUS VUMAT subroutine, successfully reproduced fracture strains and crack paths across stress states ranging from pure shear to high hydrostatic tension. Comparative simulations indicate that this approach yields improved prediction accuracy over the classical GTN model, particularly under mixed-mode conditions, thereby offering a practical numerical tool for analyzing AHSS formability.

## 1. Introduction

Advanced High-Strength Steels, such as Dual-Phase (DP) steels, offer a favorable strength–ductility balance attributed to their ferrite–martensite microstructure [1,2]. However, this microstructural heterogeneity induces local stress–strain incompatibilities during stamping, which govern a stress-state-dependent transition in fracture mechanisms [3,4]. Failure modes shift continuously from void-mediated ductile tearing at high stress triaxiality to shear-dominated localization at low triaxiality, with intermediate states exhibiting mixed characteristics.

**Figure 1:**
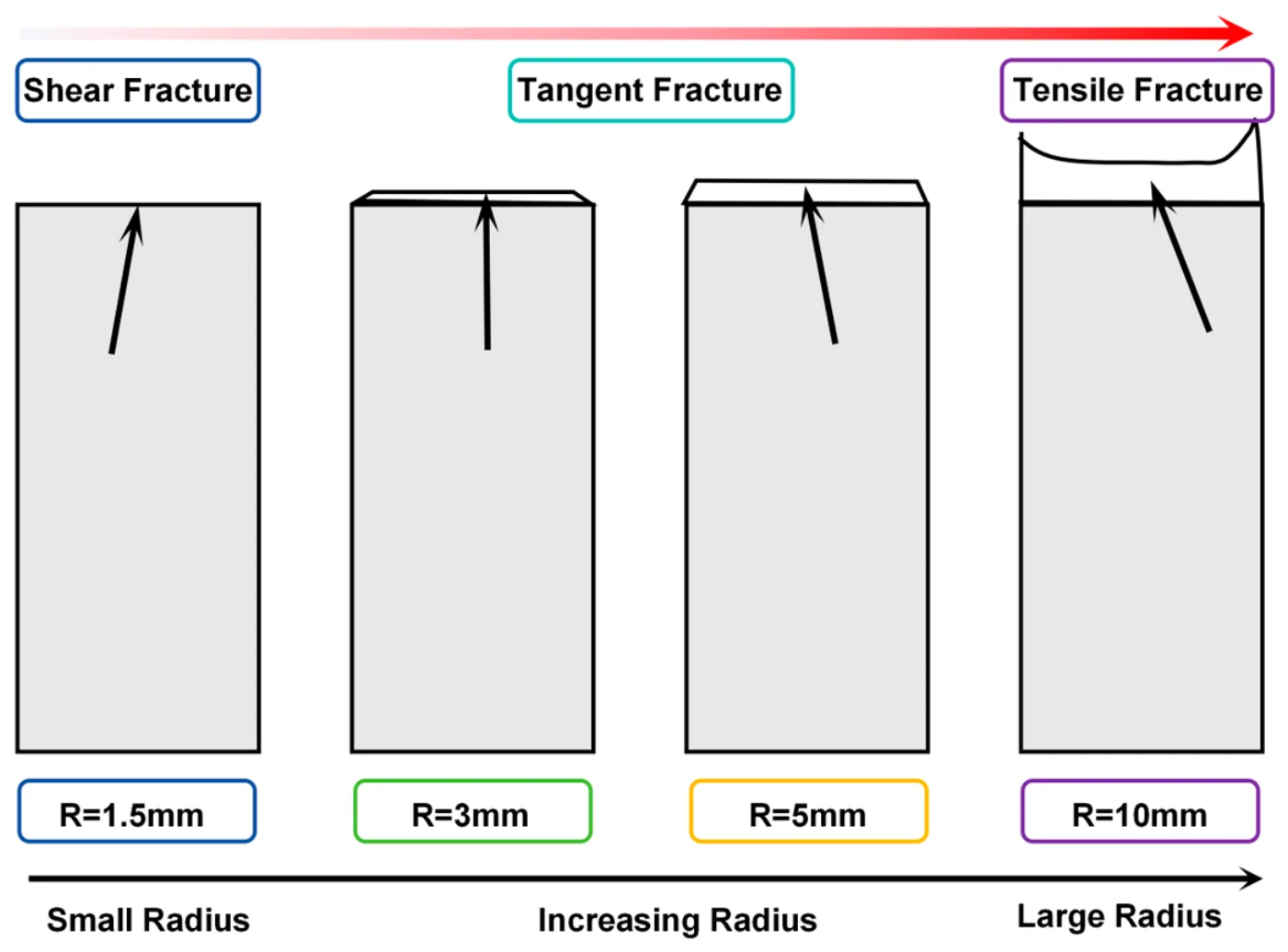
Schematic of stress-state-dependent fracture modes in tension-bending tests with varying punch radii.

In tension-bending tests, the fracture mode is governed by the punch corner radius, which controls the local stress state. As illustrated in Figure 1, small punch radii promote shear-localized fracture with minimal necking, whereas large radii favor void-mediated ductile failure characterized by extensive necking and dimple formation; intermediate radii yield mixed-mode failures [5,6]. Such sensitivity to stress state compromises the predictive accuracy of conventional Forming Limit Diagrams (FLDs) [7], which rely on necking instability, and macroscopic fracture criteria based exclusively on equivalent plastic strain accumulation.

Micromechanical damage modeling offers a viable approach to these complexities. The classical Gurson–Tvergaard–Needleman (GTN) model [8] effectively captures void-mediated ductile fracture at high stress triaxiality by tracking the void volume fraction, yet it fails to address shear-driven failure dominant at low or negative triaxiality because the damage accumulation in the GTN framework relies exclusively on volumetric plastic expansion [9,10,11,12]. Zhou et al. [13] introduced a macroscopic shear damage variable into the yield function to mitigate this limitation; however, despite its mathematical simplicity, the approach lacks explicit micromechanical grounding. Conversely, the Nahshon–Hutchinson (N-H) model [14] incorporates a deviatoric stress-dependent term directly into the void evolution equation, providing a physically based description of shear damage. The N-H shear damage increment is expressed as:

(1) $${\mathrm{df}}_{\mathrm{shear}}{=k}_{s}f\omega (\sigma_{\mathrm{ij}})\frac{s_{\mathrm{ij}}d\varepsilon_{\mathrm{ij}}^{p}}{\sigma_{eq}}$$

where df_shear_ is the incremental shear-induced damage contribution to the void volume fraction; $k_{s}$ is a material constant quantifying the sensitivity of void distortion to shear deformation; f is the current void volume fraction; ${\omega (\sigma }_{\mathrm{ij}}{)=1-\xi }^{2}$ is a stress-state weighting function in which $\xi =27J_{3}/2\sigma_{eq}^{3}$ is the normalized third invariant of the deviatoric stress tensor; $s_{\mathrm{ij}}$ is the deviatoric stress tensor; ${d\varepsilon }_{\mathrm{ij}}^{p}$ is the plastic strain increment tensor; and $\sigma_{eq}$ is the von Mises equivalent stress. The term $s_{\mathrm{ij}}{d\varepsilon }_{\mathrm{ij}}^{p}/\sigma_{eq}$ represents the normalized deviatoric plastic work increment, which quantifies the shear deformation driving void elongation and rotation within localized shear bands. Nevertheless, the N-H modification focuses primarily on damage evolution without adjusting the yield surface to reflect instantaneous shear softening [15].

Although the N-H formulation establishes a quantitative relationship between deviatoric plastic work and void distortion, stress triaxiality alone is insufficient to fully characterize the loading path. Recent studies have therefore integrated the Lode angle parameter to distinguish damage mechanisms across varying loading paths [16,17,18,19]. Current models, however, exhibit two key limitations. First, they treat void growth and shear damage as decoupled or linearly superimposed processes, thereby neglecting mesoscale synergistic interactions—most notably, void-induced shear localization. Second, they lack a unified framework driven jointly by stress triaxiality and the Lode angle parameter, and therefore cannot seamlessly capture fracture-mode transitions across the full stress-state spectrum.

Existing damage models for AHSS generally fall into two categories. Macroscopic phenomenological models [20], such as the Modified Mohr–Coulomb (MMC) model [21], prioritize computational efficiency but lack physical rigor. In contrast, micromechanical models like the GTN framework offer superior predictive accuracy by explicitly modeling micro-defect evolution.

Several modified GTN formulations have been proposed to introduce shear-damage capabilities, including the Zhou model [13] (plastic potential modification), N-H model [14] (shear damage correction), Xue model [22], MALCHE model [23], Jiang model [24], and Wu model [25].

This paper is organized as follows. In Section 2, the theoretical framework is established, briefly introducing the characterization of stress states via stress triaxiality and the Lode angle, along with the formulation of the extended GTN damage model. In Section 3, a comprehensive experimental program is presented, where shear-tension, notched tensile, and Nakazima specimens were specifically designed to cover a wide range of stress states; furthermore, scanning electron microscopy (SEM) analysis was conducted to examine fracture surfaces and identify microstructural evolution mechanisms. In Section 4, a stress-state-dependent competitive damage weight function is constructed and calibrated based on the critical transition threshold observed in micro-fractographic characteristics. In Section 5, the proposed model is validated through finite element simulations against experimental results, including stress–strain responses, fracture paths, and complex tension-bending tests, to verify its accuracy in predicting mixed-mode fractures. Finally, the main conclusions are summarized in Section 6.

## 2. Theoretical Framework

### 2.1. Stress State Parameters

Complete characterization of the stress state [26] requires two dimensionless parameters: stress triaxiality η and the normalized Lode angle $\bar{\theta }$.

The stress triaxiality η is defined as the ratio of the hydrostatic pressure $\sigma_{m}$ to the von Mises equivalent stress $\sigma_{eq}$, and quantifies the relative contribution of the tensile (or compressive) hydrostatic stress with respect to the distortional stress:

(2) $$\sigma_{m}=\frac{1}{3}\left(\sigma_{1}+\sigma_{2}+\sigma_{3}\right)$$

(3) $$s_{ij}=\sigma_{ij}-\sigma_{m}\delta_{ij}$$

(4) $$\eta =\frac{\sigma_{m}}{\sigma_{eq}}$$

where $\sigma_{1},{\;\sigma }_{2},{\;\sigma }_{3}$ are the principal stresses; $\sigma_{m}$ is the mean hydrostatic pressure; and $\sigma_{\mathrm{eq}}=\sqrt{3J_{2}}$ is the von Mises equivalent stress, with $J_{2}$ being the second invariant of the deviatoric stress tensor $s_{\mathrm{ij}}$. High η values, such as η = 1/3 in uniaxial tension, promote void nucleation and volumetric growth, leading to ductile tearing. Low or negative η values, such as η = 0 in pure shear, suppress void expansion and favor shear-band localization.

However, η alone cannot distinguish between stress states that share identical hydrostatic components but follow distinct deviatoric loading paths. For example, axisymmetric tension and plane-strain tension may both yield η = 1/3, yet their effects on damage evolution differ markedly. To resolve this ambiguity, the Lode angle is introduced through the second and third invariants of the deviatoric stress tensor:

(5) $$J_{2}=\frac{1}{2}s_{ij}s_{ij},\;\;J_{3}=det\left(s_{ij}\right)$$

where $s_{\mathrm{ij}}s_{\mathrm{ij}}$ denotes the sum of squares of all components of the deviatoric stress tensor, and $\det\left(s_{\mathrm{ij}}\right)$ is the determinant of the deviatoric stress tensor matrix. $J_{2}$ quantifies the magnitude of the deviatoric stress and $J_{3}$ characterizes its asymmetry in the π-plane. The Lode angle $\theta \in \left[0,\pi /3\right]$ relates to these invariants by:

(6) $$cos\left(3\theta \right)=\frac{3\sqrt{3}}{2}\frac{J_{3}}{{J_{2}}^{3/2}}=\frac{27J_{3}}{2{\sigma_{eq}}^{3}}$$

For practical use, this study adopts the normalized Lode angle parameter $\theta \in \left[-1,1\right]$, obtained by linearly mapping θ:

(7) $$\theta =\frac{1}{3}arccos\frac{27J_{3}}{2{\sigma_{eq}}^{3}}$$

(8) $$\bar{\theta }=1-\frac{6}{\pi }\theta$$

**Figure 2:**
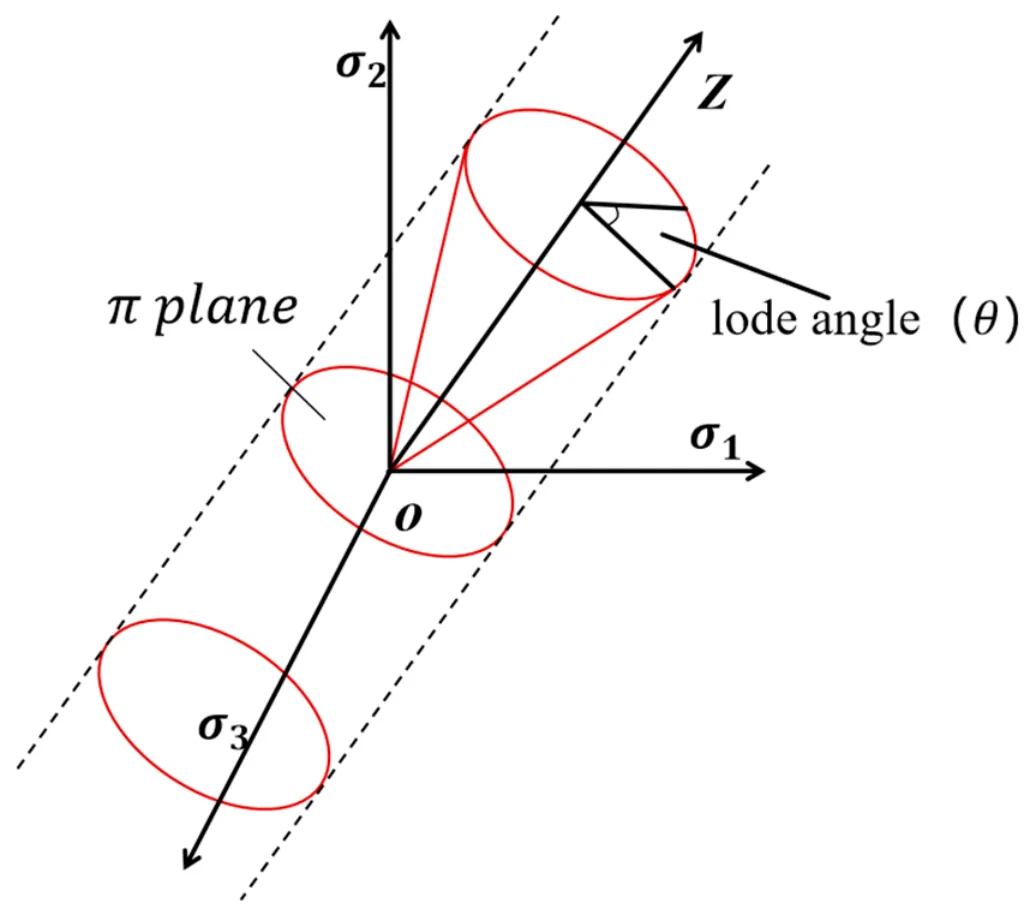
Principal stress space showing the hydrostatic axis, the π-plane, and the geometric definition of the Lode angle parameter θ.

The geometric interpretation of $\bar{\theta }$ is illustrated in Figure 2. In the principal stress space, the hydrostatic axis represents states of equal principal stresses ($\sigma_{1}=\sigma_{2}=\sigma_{3}$). The plane perpendicular to this axis is the π-plane, onto which the deviatoric stress state is projected. The normalized Lode angle parameter $\bar{\theta }$ describes the angular position of this projection within the π-plane. Physically, $\bar{\theta }=-1$ corresponds to axisymmetric compression or plane-strain tension; $\bar{\theta }=1$ corresponds to axisymmetric tension; and $\bar{\theta }=0$ corresponds to pure shear. Together, η and $\bar{\theta }$ uniquely determine any stress state in the principal stress space.

### 2.2. Extended GTN Damage Evolution Model

In the extended GTN framework [27], the evolution of the void volume fraction is governed by a stress-state-dependent weighting function $\beta (\eta ,\;\bar{\theta }\;)$, which continuously partitions the total damage increment between two competing mechanisms. The total increment is expressed as:

(9) $${\mathrm{df}=\beta (\eta ,\;\bar{\theta }\;)(\mathrm{df}}_{\mathrm{gr}}{+\mathrm{df}}_{\mathrm{nucl}}{)+[1-\beta (\eta ,\;\bar{\theta }\;)]\mathrm{df}}_{\mathrm{shear}}$$

where df is the total increment of the void volume fraction; $\left(\eta ,\;\bar{\theta }\;\right)$ is the stress-state-dependent competing weight function; ${\mathrm{df}}_{\mathrm{gr}}$ is the void growth increment driven by plastic incompressibility; ${\mathrm{df}}_{\mathrm{nucl}}$ is the void nucleation increment following a Gaussian distribution; and ${\mathrm{df}}_{\mathrm{shear}}$ is the shear damage increment described by the N-H formulation (Equation (1)). The weighting function β dynamically partitions the total damage between these two mechanisms based on the local stress state defined in Section 2.1; its specific functional form is constructed in Section 4.3.

## 3. Experiments

### 3.1. Material and Specimen

**Figure 3:**
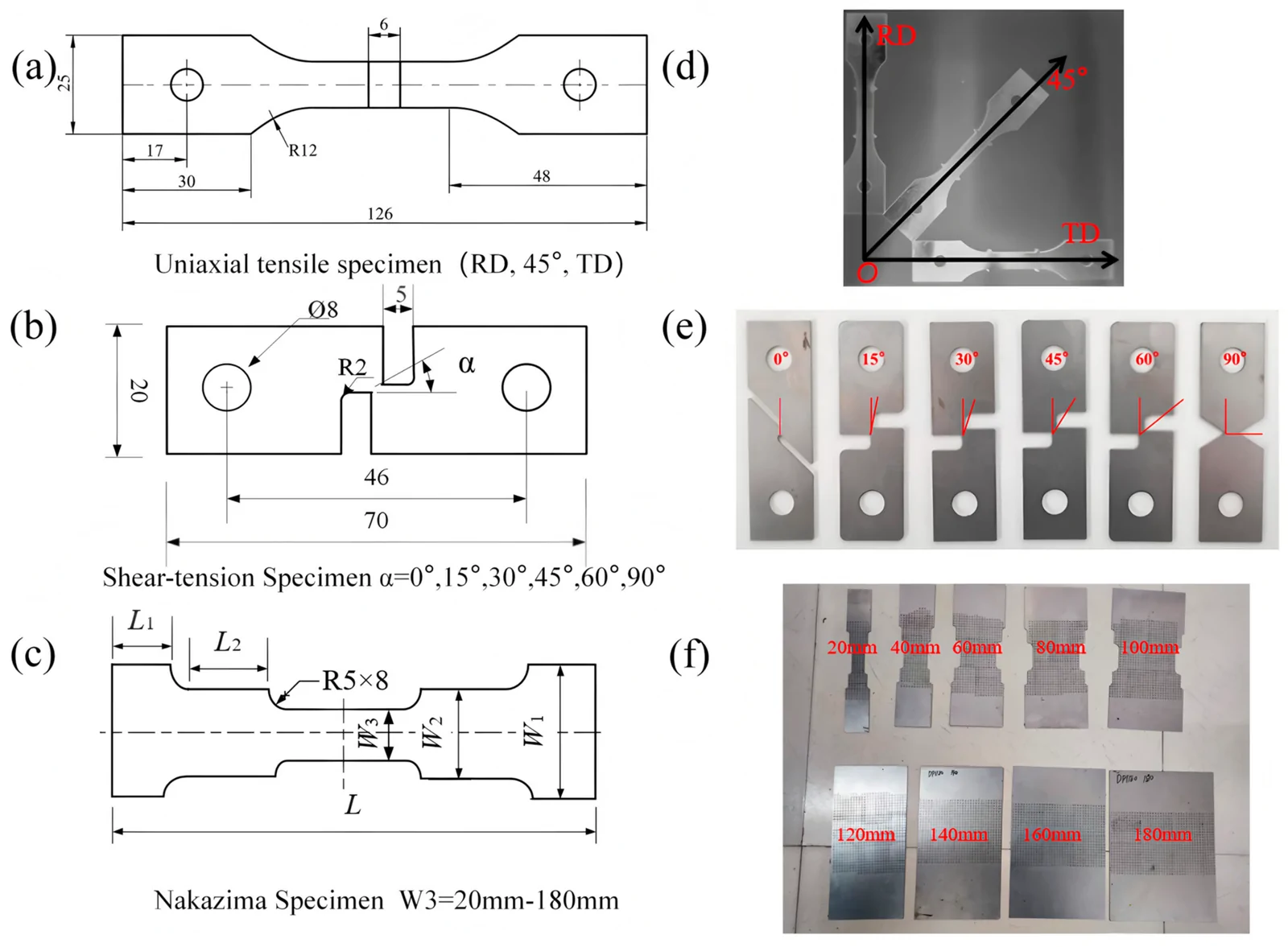
Schematic diagram and fabricated samples of the test specimens: Unit: mm; (**a**) tension; (**b**) shear-tension; (**c**) Nakazima; (**d**) directional layout; (**e**) shear-tension series; (**f**) Nakazima series.

To characterize the stress-state-dependent fracture of DP1180 steel, experiments were performed on a 1.0 mm thick commercial cold-rolled sheet. Table 1 lists the material’s chemical composition. Uniaxial tensile specimens were cut from a single sheet along the rolling, diagonal, and transverse directions to ensure consistency and to provide the directional data required for Hill’48 constitutive calibration. Figure 3a shows the ASTM E8-compliant dog-bone specimens used for that calibration.

By combining six new shear-tension specimens [28] with the Nakazima test [29], the test matrix covers a wide range of stress triaxiality and normalized Lode angle. The shear angle α, defined as the angle between the notch ligament and the loading direction, was varied from 0° to 90° in 15° increments. Center-notched specimens with different notch radii and shear angles were fabricated by wire-EDM (Figure 3b,e), allowing normal and shear loading components to be combined in the notch region and thereby enabling controlled modification of the local stress state and fracture path.

Complementing the shear-tension series, Nakazima specimens with central widths from 20 mm to 180 mm (Figure 3c,f) were used to access high-triaxiality regimes under varying normalized Lode angle. The two specimen families therefore produce partially overlapping $\left(\eta ,\;\bar{\theta }\;\right)$ coverage: the shear-tension series populates low-to-intermediate triaxiality, whereas the Nakazima series extends toward biaxial tensile states. Surface grids were etched on all forming specimens to enable local strain measurement via digital image correlation, providing the strain data required to determine fracture initiation and the corresponding stress-state history.

### 3.2. Experimental Procedure

**Figure 4:**
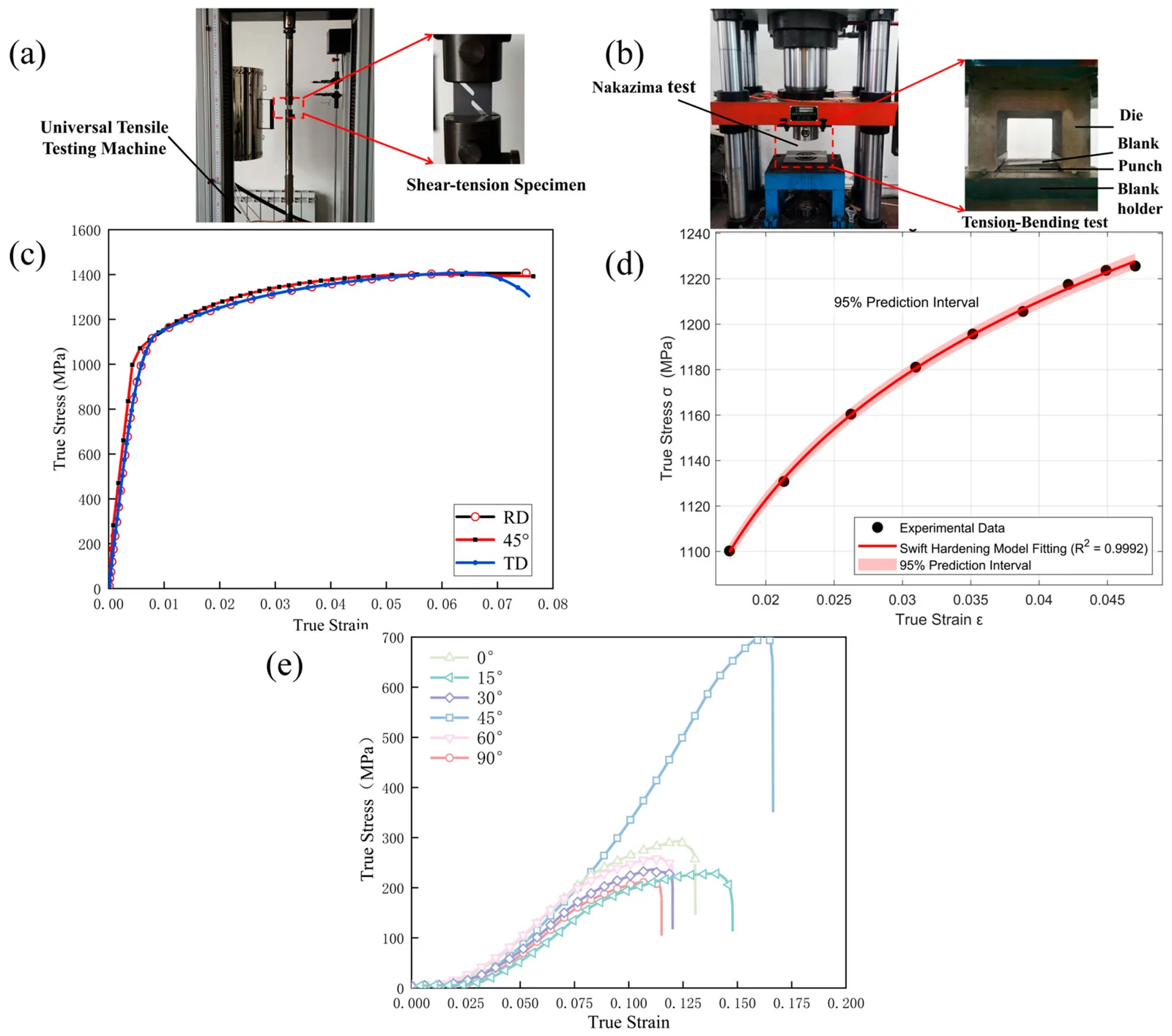
Experimental true stress–strain responses and model fitting results: (**a**) uniaxial tensile setup; (**b**) Nakazima biaxial testing system; (**c**) directional true stress–strain curves (RD, 45°, TD); (**d**) Swift hardening law fitting; (**e**) notched specimen true stress–strain curves for damage calibration.

Uniaxial and notched tensile tests were conducted on a WDW-20D electronic universal testing machine under displacement control at a constant crosshead speed of 1.0 mm/min, corresponding to an initial engineering strain rate of approximately $3.3\times 10^{-4}s^{-1}$, with axial strains tracked by a non-contact video extensometer, as shown in Figure 4a. For biaxial loading, Nakazima tests were performed at a punch displacement rate of 1.5 mm/s using a hydraulic press system, illustrated in Figure 4b. True stress–strain curves were derived from force–displacement data prior to necking. Using tensile data from the rolling direction (RD), the 45° direction, and the transverse direction (TD), the Hill’48 yield criterion and Swift hardening law were calibrated via least-squares regression. Table 2 lists the resulting anisotropic and hardening parameters, which reproduce the experimental yield stresses and plastic strain ratios with deviations below 5%. Figure 4e presents the true stress–strain curves for the notched specimens, yielding the local strain values required to calibrate the damage initiation criteria discussed in Section 4.

### 3.3. Analysis of Experimental Data

#### 3.3.1. Stress State Distribution

**Figure 5:**
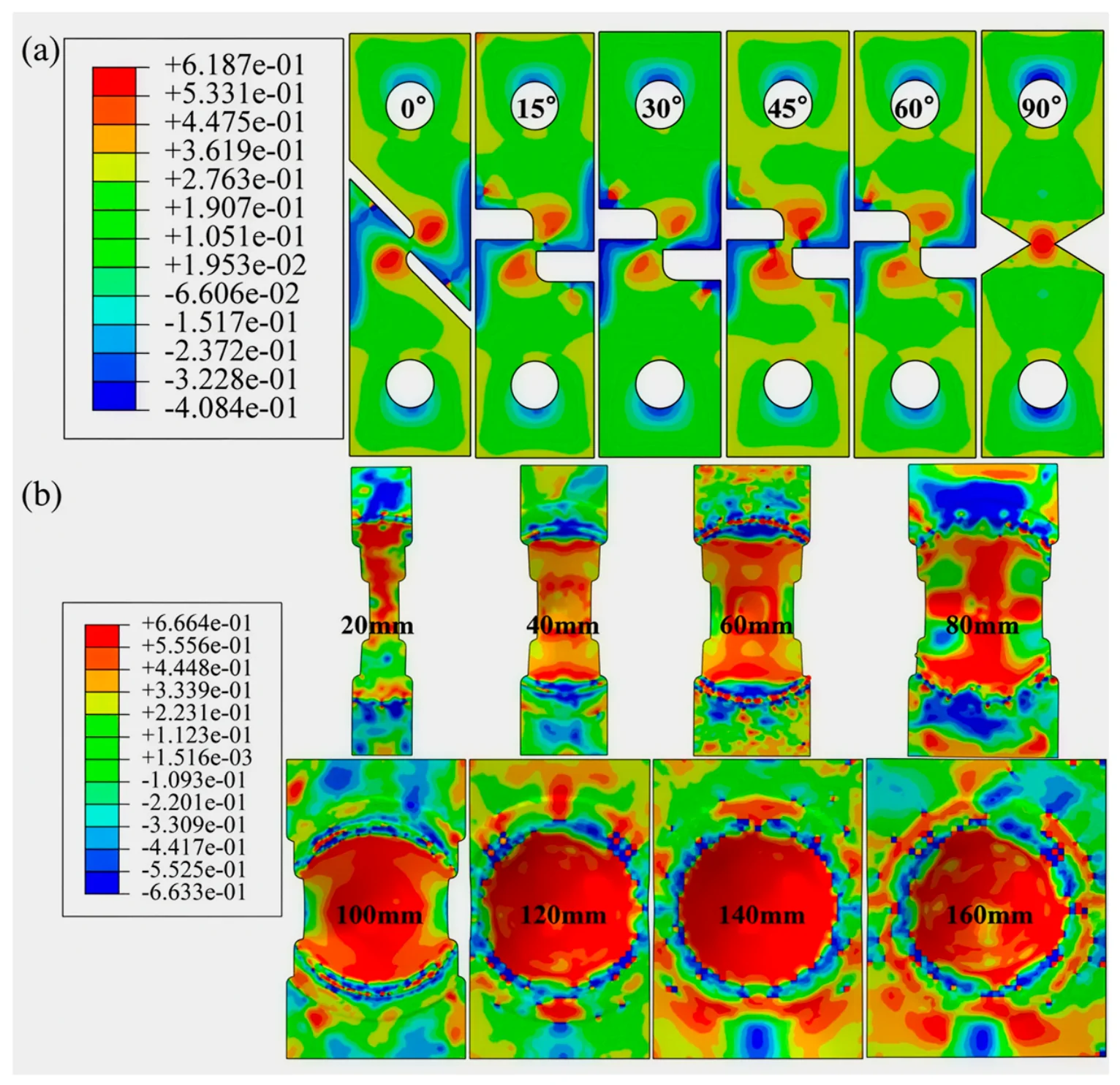
Simulated stress triaxiality distributions in specimens of varying sizes: (**a**) shear-tension specimens; (**b**) Nakazima specimens.

Finite element simulations indicate that stress states evolve with specimen geometry. Figure 5 illustrates how increasing the shear angle from 0° to 90° shifts the minimum stress triaxiality from pure shear to tensile states, while Nakazima specimens maintain η > 0.5 across the uniaxial-to-biaxial transition.

#### 3.3.2. Fracture Strain and Stress Triaxiality

The fracture equivalent plastic strain $\varepsilon_{f}$ is the equivalent plastic strain at the instant of macroscopic fracture. It is determined by identifying the fracture point on the true stress–strain curve—the data point immediately preceding the abrupt stress drop—and subtracting the elastic strain component from the total true strain at that point. The resulting $\varepsilon_{f}$ value is then used to locate the corresponding instant in the finite element simulation, from which the critical integration point and its stress-state history are extracted.

**Figure 6:**
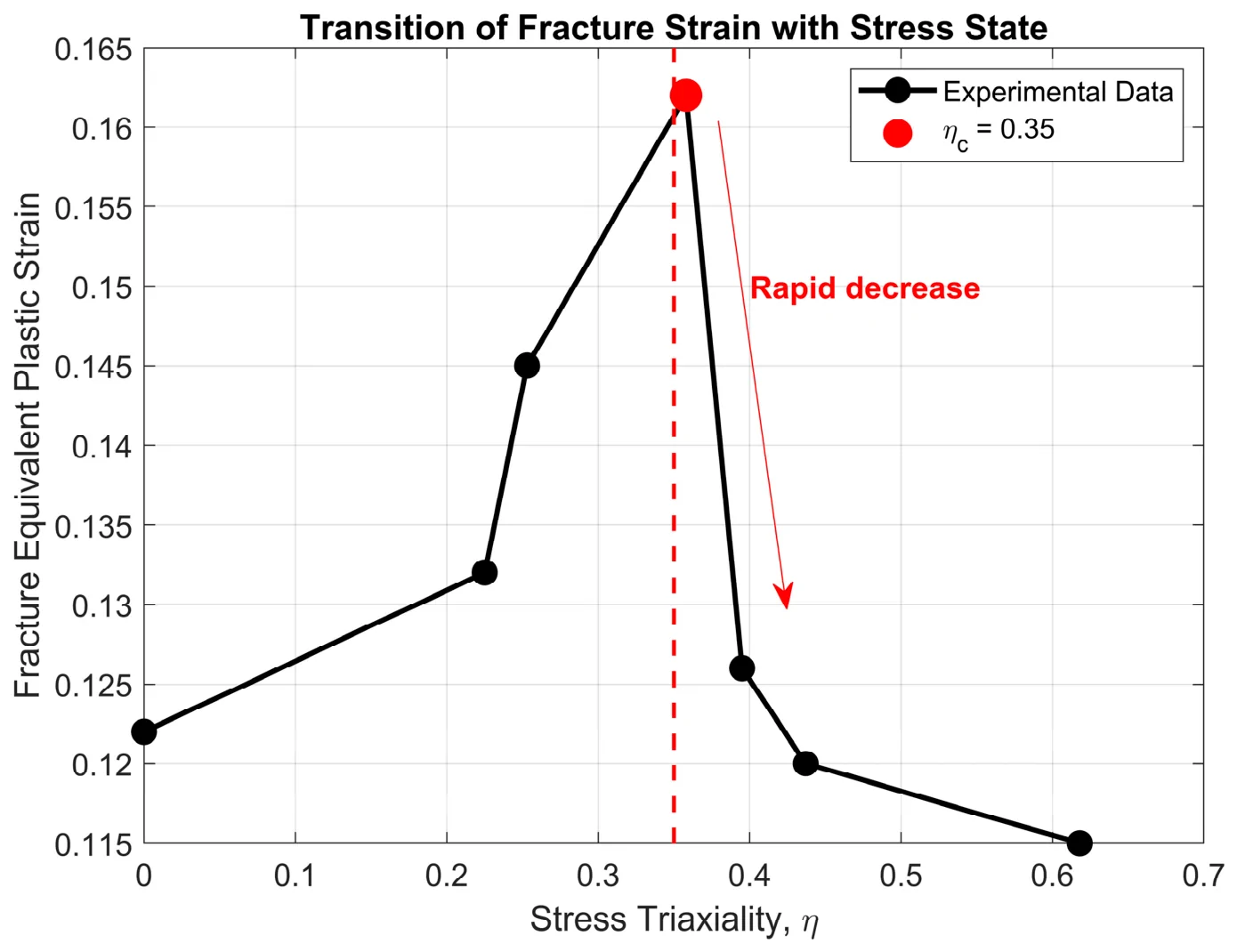
Fracture strains and associated stress states derived from notched tensile tests.

Because the stress state at the critical point evolves continuously during loading, a single instantaneous value of the stress triaxiality cannot adequately represent the loading path. We therefore compute an average stress triaxiality $\eta_{avg}$ for each specimen using the strain-weighted integration:

(10) $$\eta_{avg}=\frac{1}{\varepsilon_{f}}\int_{0}^{\varepsilon_{f}}\eta \left({\bar{\varepsilon }}^{p}\right)d{\bar{\varepsilon }}^{p}$$

where $\eta \left({\bar{\varepsilon }}^{p}\right)$ is the instantaneous triaxiality history extracted from the critical integration point, and ${\bar{\varepsilon }}^{p}$ is the accumulated equivalent plastic strain. This averaging accounts for non-proportional loading effects and assigns greater weight to strain intervals in which more damage accumulates. The fracture strains $\varepsilon_{f}$ for the 0–90° notched specimen series are plotted against their corresponding $\eta_{avg}$ in Figure 6. The resulting relationship exhibits a pronounced non-monotonic trend. For $\eta_{avg}$ < 0.35, $\varepsilon_{f}$ increases with triaxiality: moderate hydrostatic tension suppresses premature shear-band localization in the ferrite matrix, thereby delaying fracture. At $\eta_{avg}$ ≈ 0.35, $\varepsilon_{f}$ reaches a local maximum of approximately 0.162. For $\eta_{avg}$ > 0.35, $\varepsilon_{f}$ drops sharply as elevated hydrostatic tension promotes rapid void nucleation at martensite–ferrite interfaces and accelerates void growth and coalescence. The triaxiality at this local maximum is identified as the critical stress triaxiality. Physically, $\eta_{c}$ marks the transition from shear-dominated fracture to void-dominated fracture.

#### 3.3.3. Influence of Normalized Lode Angle

**Figure 7:**
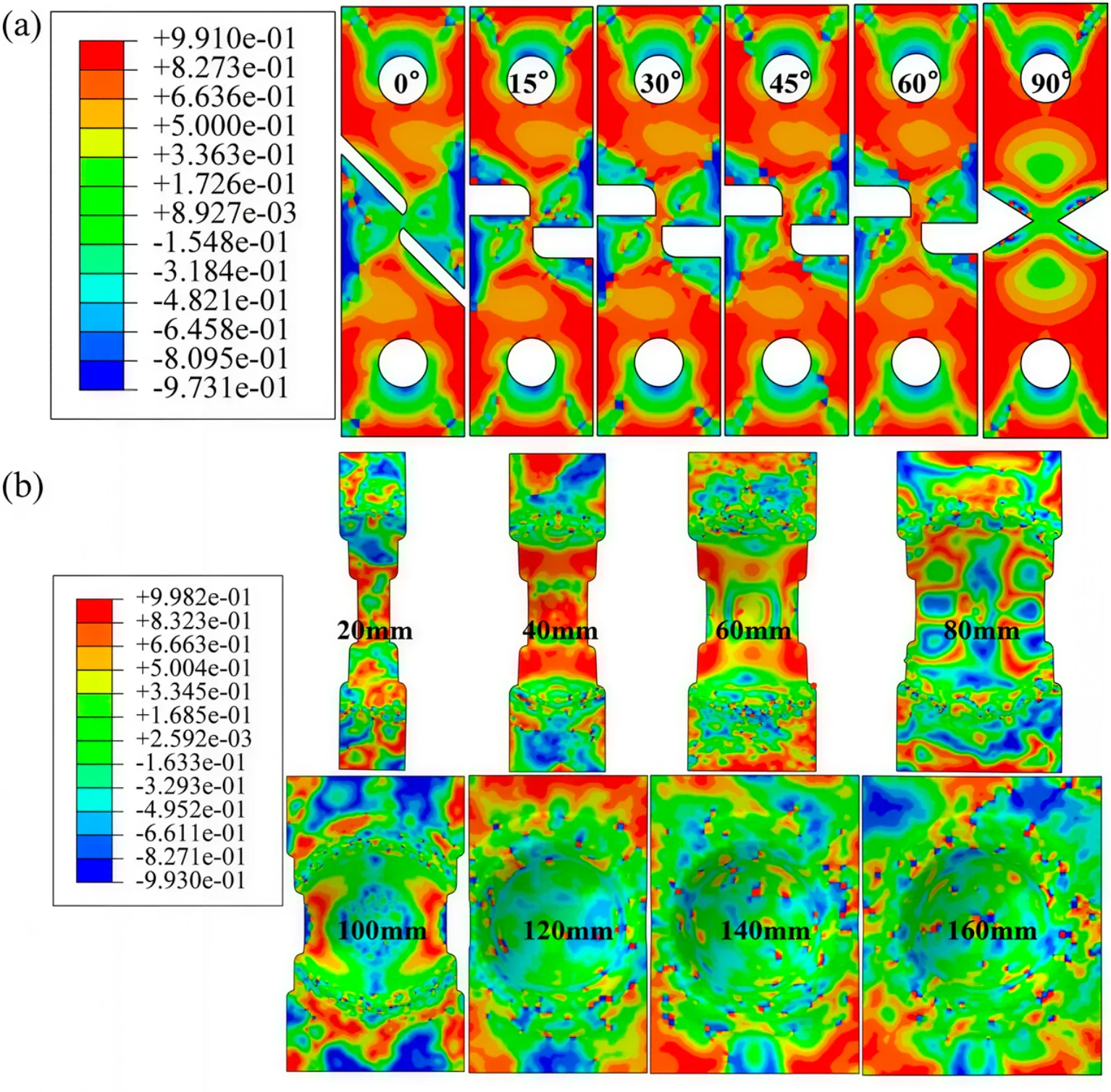
Simulated normalized Lode angle distributions in specimens of varying sizes: (**a**) shear-tension specimens; (**b**) Nakazima specimens.

**Figure 8:**
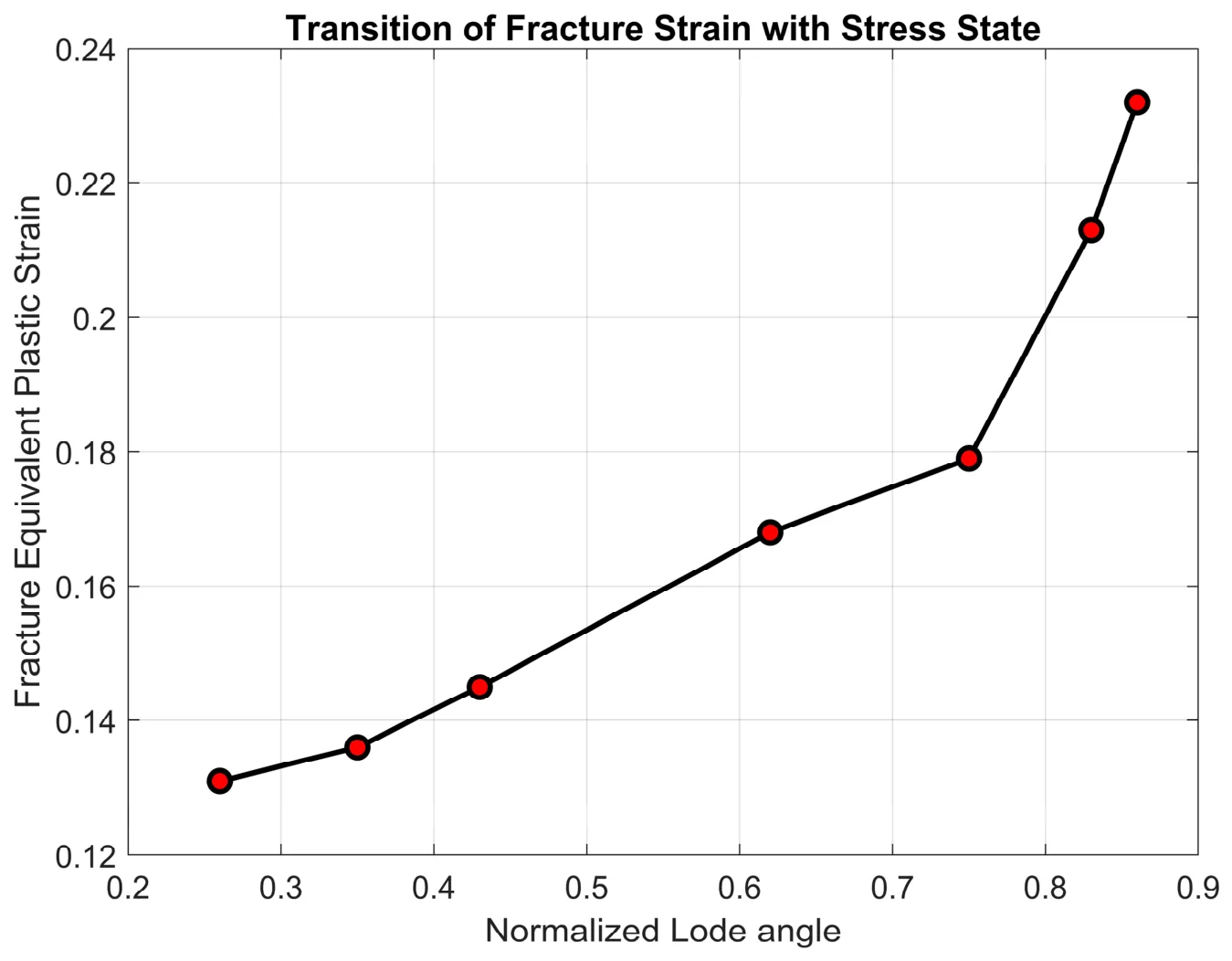
Dependence of fracture strain on the normalized Lode angle.

Stress triaxiality alone cannot fully describe fracture behavior. Figure 7 and Figure 8 demonstrate that varying the normalized Lode angle significantly alters ductility under similar triaxiality conditions; lower angles accelerate failure via shear damage, whereas higher angles stabilize void growth. These findings support the adoption of a dual-parameter (η, $\;\bar{\theta }$) damage framework.

## 4. Construction of the Competitive Damage Weight Function

### 4.1. Micro-Fractographic Characterization

**Figure 9:**
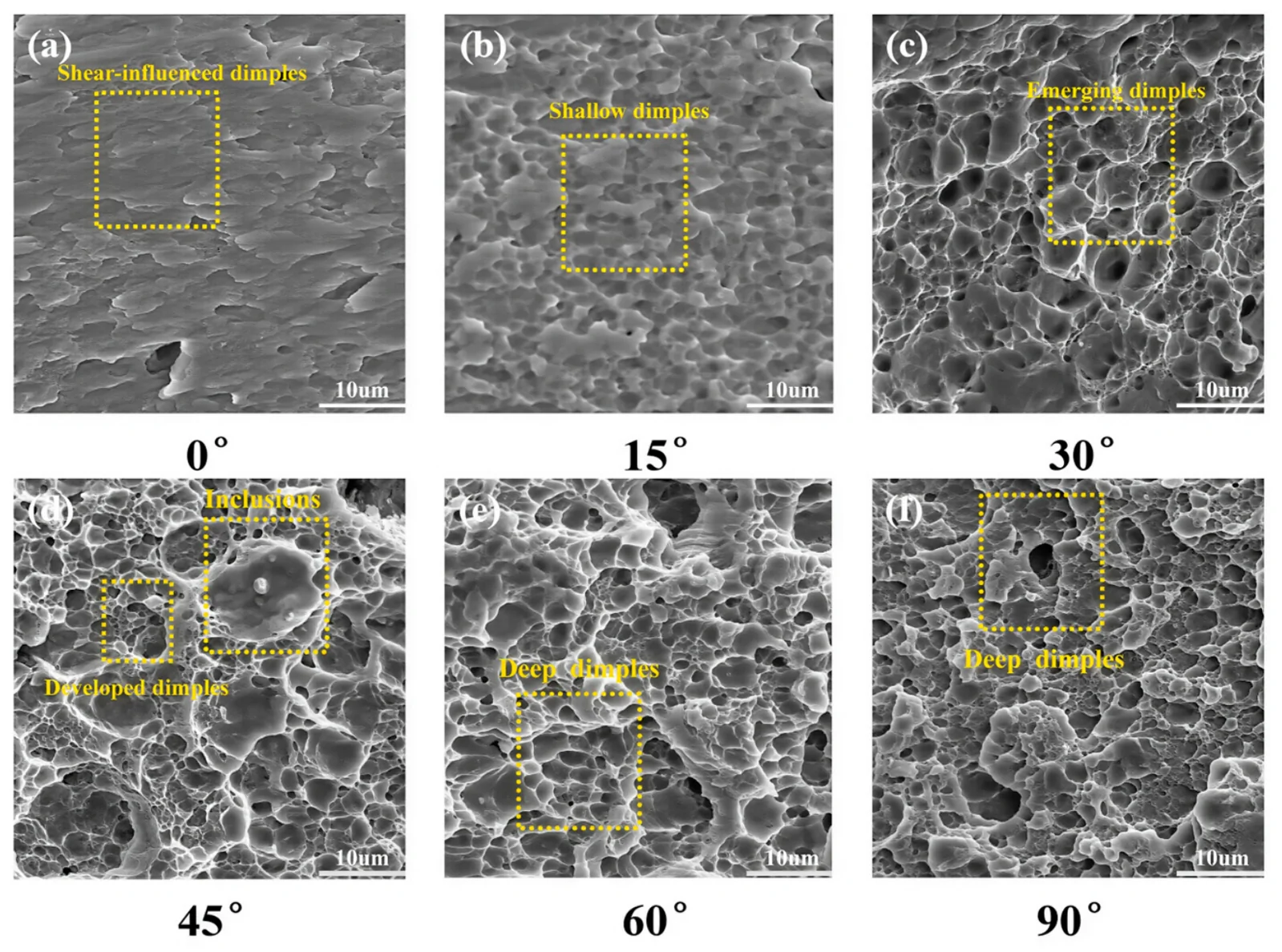
(**a**–**c**) Shear-dominated fracture; (**d**) mixed-mode; (**e**,**f**) void-dominated fracture with deep dimples.

Scanning electron microscopy (SEM) analysis revealed the correspondence between macroscopic fracture modes and underlying micro-mechanisms. Figure 9 shows that shear-dominated fracture features tearing ridges with sharp tips and severely elongated dimples on flat facets, suggesting micro-void sliding and rupture within adiabatic shear bands. Conversely, void-dominated fracture exhibits deep, equiaxed dimples resulting from particle debonding and hydrostatic tension. Mixed-mode specimens display a coexistence of tearing ridges and dimples, indicating a competition between these mechanisms. As stress triaxiality increases, the fracture morphology evolves continuously from shear-induced elongated dimples at low triaxiality to tension-driven equiaxed voids at high triaxiality, as compiled in Figure 9.

**Figure 10:**
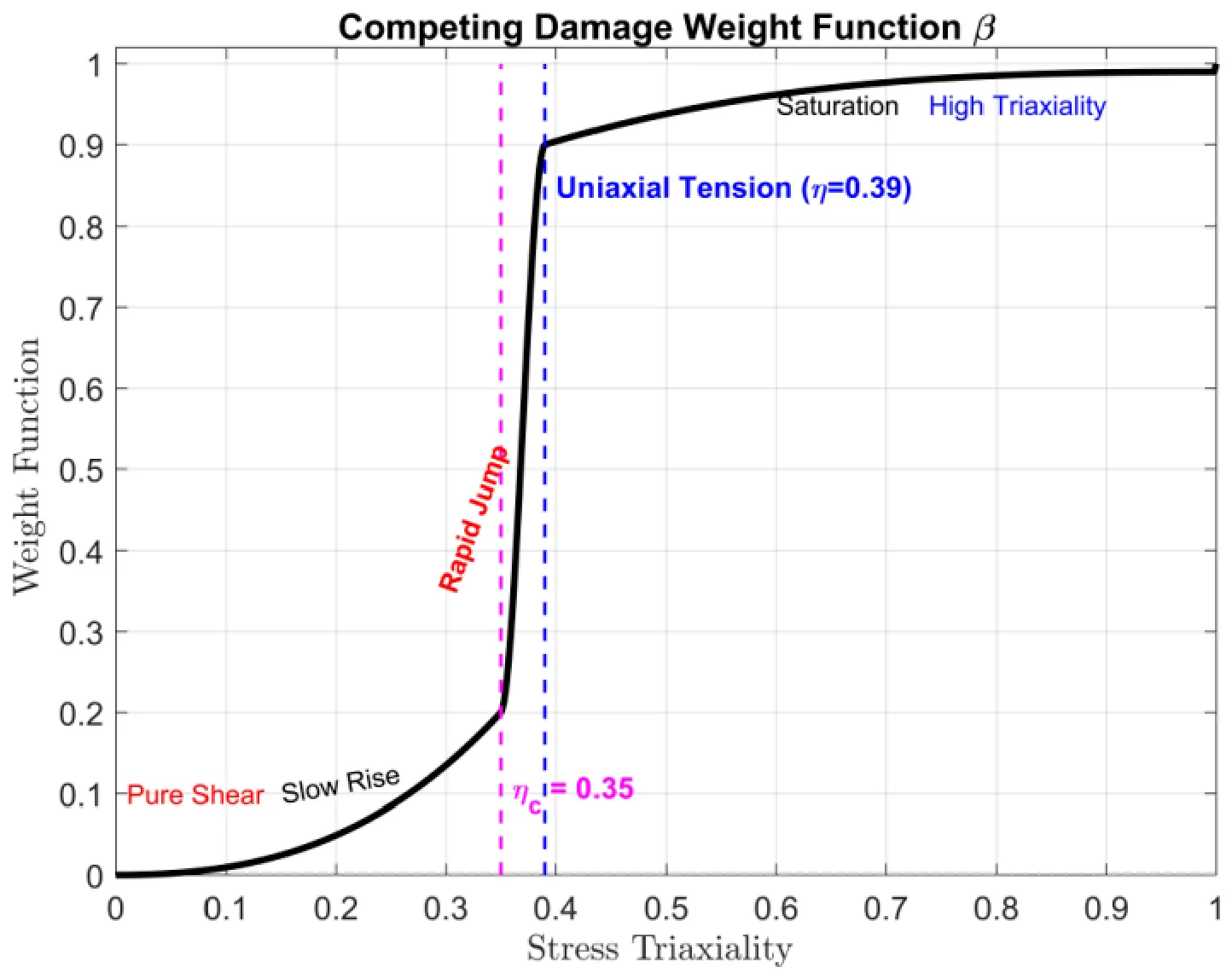
Schematic of the weight function governing competitive damage mechanisms.

These fractographic observations directly motivate the conceptual model illustrated in Figure 10. The SEM evidence demonstrates that the dominant damage mechanism is not a material constant but a function of the local stress state: shear features prevail at low triaxiality, equiaxed dimples dominate at high triaxiality, and both coexist in the intermediate range. To encode this continuous transition into the constitutive framework, we adopt the sigmoid (logistic) function as the mathematical basis of the competing weight function. The standard sigmoid function is given by:

(11) $$S\left(x\right)=\frac{1}{1+\exp(-x)}$$

This function possesses three properties that make it well suited to describing the observed fracture-mode transition. First, it is bounded between two horizontal asymptotes: $S\left(x\right)$→0 as x→−∞ and $S\left(x\right)$→1 as x→+∞, which naturally correspond to the two limiting damage mechanisms—pure shear and pure void growth, respectively. Second, it is strictly monotonic and continuously differentiable over its entire domain, ensuring that the damage-mode transition occurs without numerical discontinuities that could destabilize the finite-element solution. Third, the transition steepness is controlled by a single scaling factor in the exponent, providing a physically interpretable calibration parameter. These characteristics align closely with the SEM observations in Figure 9: the shear-to-void transition is not an abrupt switch but a smooth, continuous evolution spanning a finite range of stress triaxiality centered near the critical value $\eta_{c}$ ≈ 0.35.

### 4.2. Calibration of Critical Stress Triaxiality

**Figure 11:**
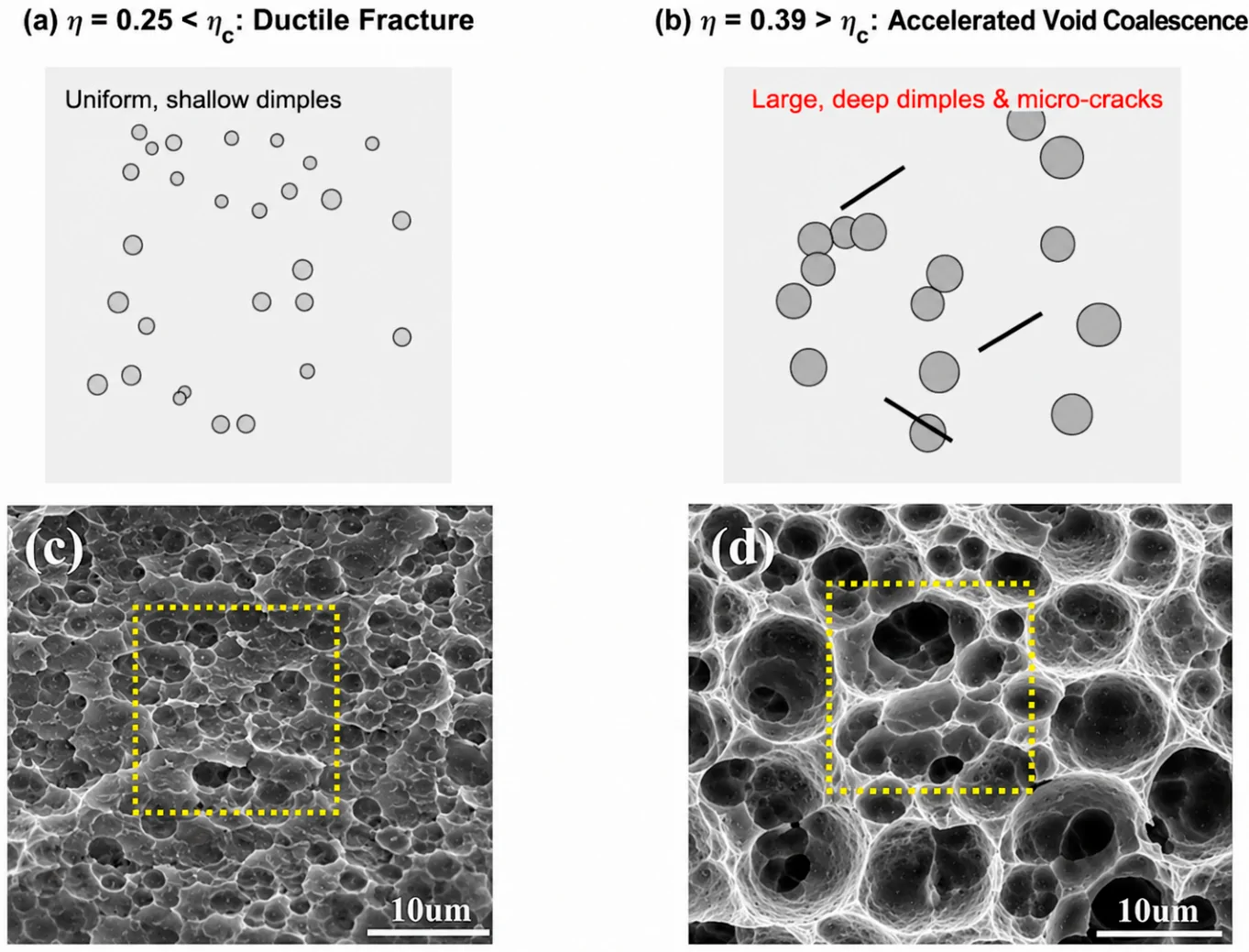
(**a**) uniformly distributed shallow dimples; (**b**) enlarged voids with micro-crack formation; (**c**) fine and homogeneous dimple structure observed by SEM; (**d**) coarse dimple morphology associated with developed void structures observed by SEM.

The quantitative SEM analysis of the shear-tension specimens linked the dominant micro-mechanism to the local stress state. The void-dominated area fraction and the average void diameter were measured as functions of stress triaxiality η. As η increased from about 0.25 to 0.39, the void-dominated damage fraction rose monotonically from 10% to 90%, while the average void diameter increased sharply, as shown in Figure 11. This continuous transition indicates a shift from shear-dominated to void-dominated damage within a limited triaxiality interval. The transition threshold gives a critical stress triaxiality of $\eta_{c}$ = 0.35.

### 4.3. Competitive Damage Function

**Figure 12:**
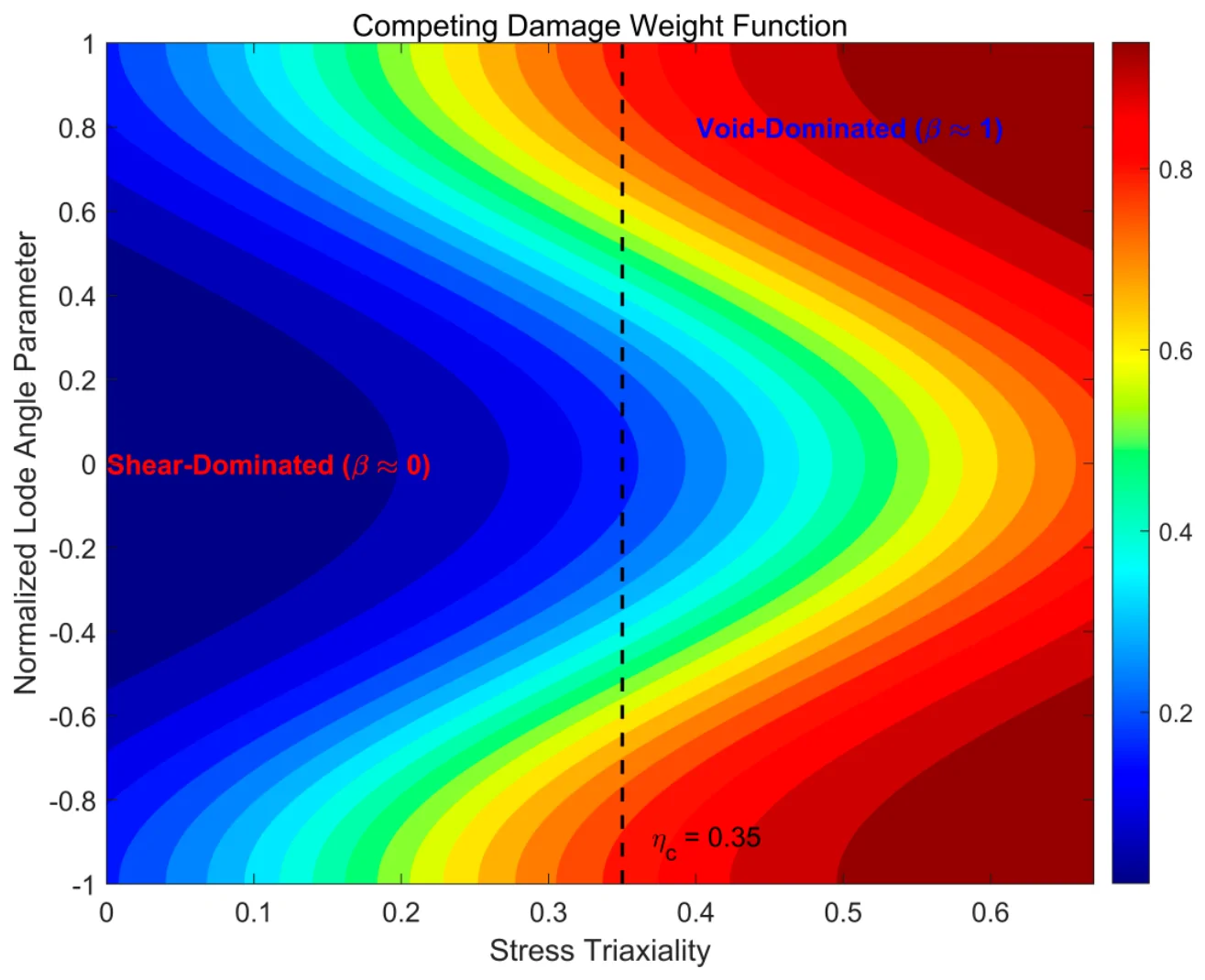
Weighting coefficients describing the competition between ductile and shear fracture.

To embed the dual stress state dependence identified in Section 4.1, the scalar argument x in the standard sigmoid is replaced by a linear combination of the stress triaxiality η and the normalized Lode angle $\;\bar{\theta }$. The resulting competing weight function is expressed as:

(12) $$\beta \left(\eta ,\;\bar{\theta }\right)=\frac{1}{1+\exp\left[a_{1}\left(\eta_{c}-\eta \right)-\cos\left(3\;\bar{\theta }\right)\right]}$$

where parameters $a_{1}$ and $a_{2}$ govern the transition sharpness along the triaxiality axis and the shift of the transition boundary with the normalized Lode angle parameter, respectively. Physically, β→0 indicates that shear-band-driven fracture dominates; β→1 indicates that void-coalescence-driven fracture dominates. The sigmoid form ensures continuous, differentiable transition without artificial discontinuities, ensuring physical consistency between pure shear and high triaxiality limits. For DP1180 steel, the critical threshold is $\eta_{c}$ = 0.35. Figure 12 validates this model by comparing predicted damage weights against experimental observations, showing close alignment across all stress states.

## 5. Model Validation

### 5.1. Parameter Calibration

A hierarchical calibration strategy was employed. The classical GTN constitutive parameters were fixed at their established literature values: $q_{1}$ = 1.5, $q_{2}$ = 1.0, and $q_{3}$ = 2.25 [30,31,32], where $q_{1}$ and $q_{2}$ are the Tvergaard correction coefficients that modify the void–matrix interaction in the yield function, and $q_{3}$ = ${q_{1}}^{2}$ accounts for the accelerated loss of load-carrying capacity during void coalescence. The void nucleation parameters were adopted from prior studies on dual-phase steels: $\varepsilon_{N}$ = 0.3 is the mean equivalent plastic strain at which 50% of the available void-nucleating particles are activated, controlling the onset of strain-controlled nucleation; $S_{N}$ = 0.1 is the standard deviation of the Gaussian nucleation strain distribution, governing the range of plastic strain over which new voids appear; and $f_{n}$ = 0.018 is the volume fraction of nucleating second-phase particles, determining the saturation level of the nucleation process. The initial void volume fraction was set to f0 = 0.0002, reflecting the low initial porosity of the as-received cold-rolled sheet. The critical and final void volume fractions—$f_{c}$ = 0.023 and $f_{f}$ = 0.1, respectively—define the onset of void coalescence and the complete loss of material load-carrying capacity.

Conversely, the competing weight function parameters a1 and a2, along with the Nahshon–Hutchinson shear coefficient $k_{\omega }$, were determined via an inverse finite element method. Finite element models replicating the experimental geometries and boundary conditions were optimized against global force–displacement responses and local fracture strains from smooth and notched specimens. An iterative algorithm minimized the residuals between the simulated and experimental data to derive the optimal parameter set, as summarized in Table 3.

### 5.2. Simulation Validation

#### 5.2.1. Uniaxial Tensile Response

**Figure 13:**
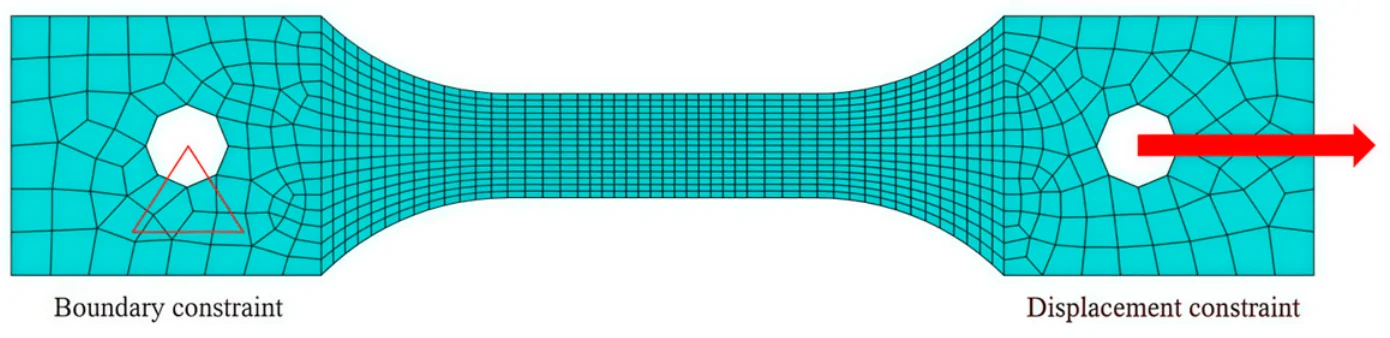
Finite element mesh of the dog-bone specimen subjected to uniaxial tension.

The constitutive model was implemented in ABAQUS/Explicit via a VUMAT subroutine. As illustrated in Figure 13, the uniaxial tensile specimens were discretized using four-node S4R shell elements, with a mesh convergence study determining an optimal element size of 0.8 mm. To replicate experimental conditions, displacement loads were applied at reference points coincident with the pin centers.

**Figure 14:**
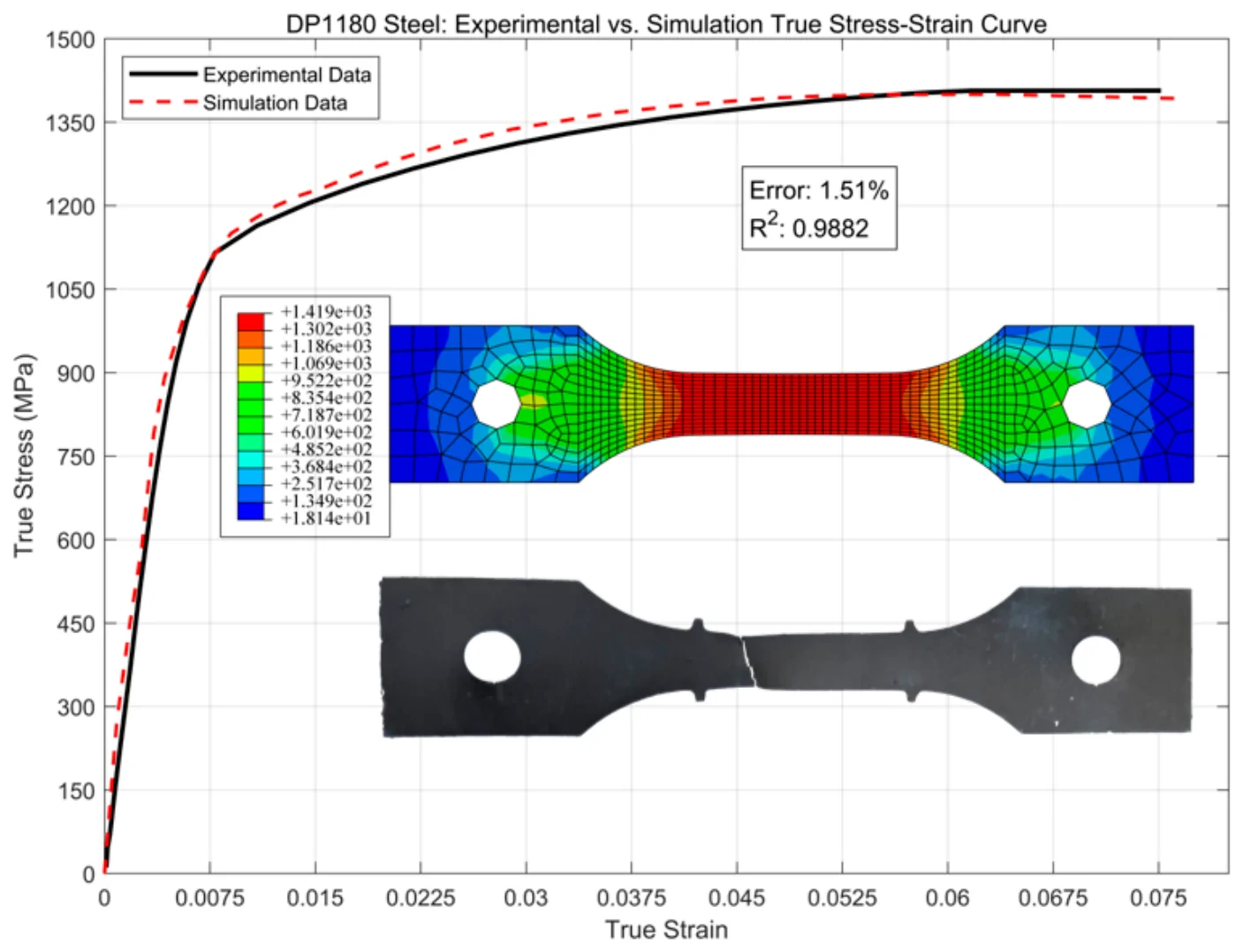
Validation of the simulated stress–strain response against experimental data.

The constitutive model was validated by comparing simulated true stress–strain curves against experimental data. Discrepancies were quantified using relative error δ and standard deviation S, governed by Equations (13) and (14):

(13) $$\delta =\frac{|\sigma^{\prime }-\sigma |}{\sigma }\times 100\%$$

(14) $$S=\sqrt{\frac{1}{N}\overset{N}{\underset{i=1}{\sum }}{{(\Delta \sigma }_{i}^{\prime }-\mu )}^{2}},\;i=1,2,3\cdots$$

where $\sigma^{\prime }$ is the simulated stress, σ is the experimental stress, μ is the mean of ${\Delta \sigma }_{i}^{\prime }=\sigma_{i}^{\prime }-\sigma_{i}$, and N is the number of data points. As depicted in Figure 14, the simulations align well with the experiments across all tested conditions. With standard deviations remaining below 8% for all specimens, the model effectively captures the anisotropic stress–strain response of DP1180 steel.

#### 5.2.2. Fracture Path and Damage Evolution

**Figure 15:**
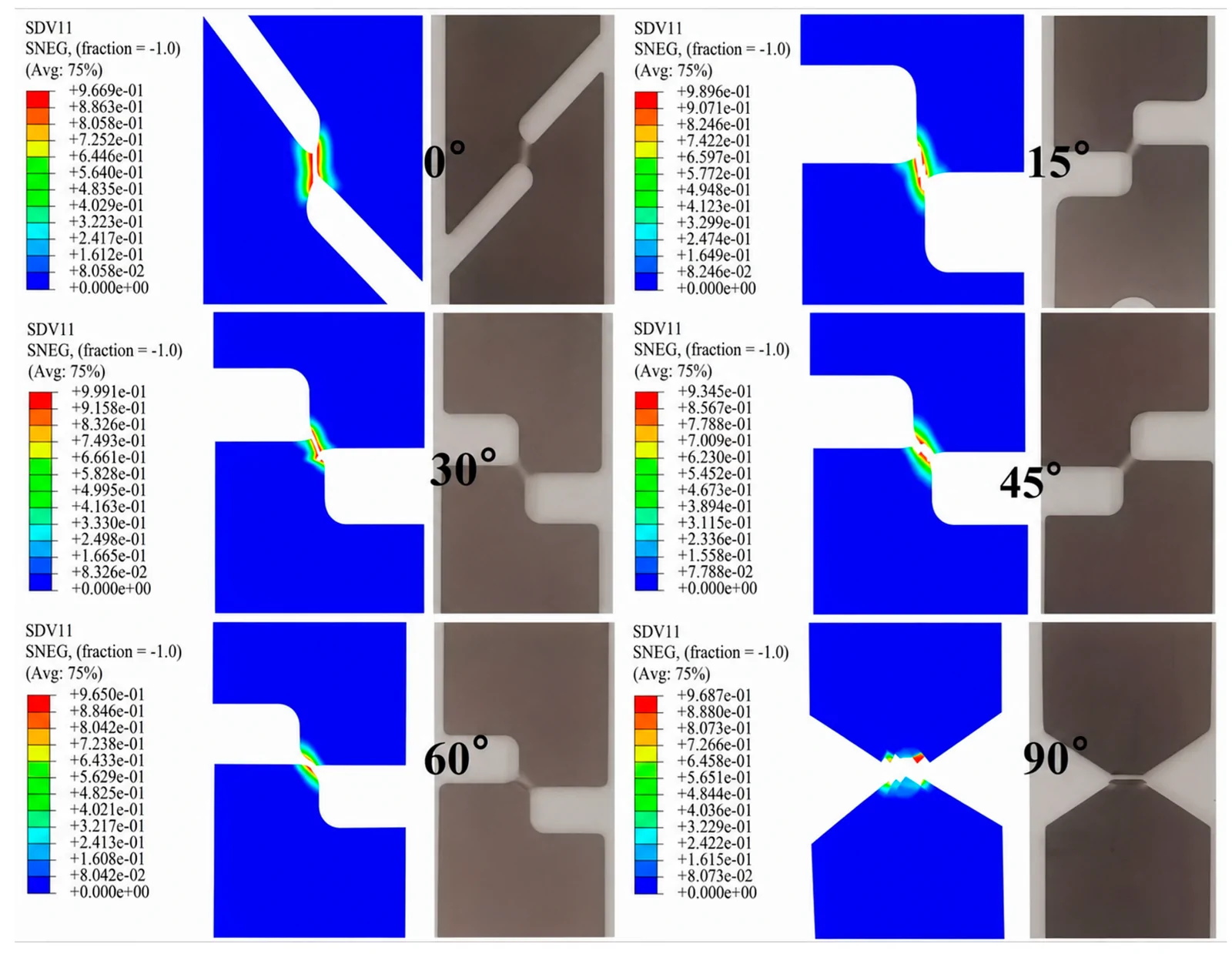
Evolution of crack trajectories with increasing stress triaxiality.

Total damage variables were normalized within the subroutine to facilitate visualization. As illustrated in Figure 15, the simulated fracture paths for notched specimens align well with the experimental observations across varying stress triaxialities. At low triaxiality, exemplified by the 0° shear specimen, the model predicts straight crack propagation along the central ligament, consistent with the observed flat shear fracture. Increasing the notch angle shifts the crack path toward the notch root, while high triaxiality induces damage localization at the narrowest cross-section accompanied by significant necking, matching experimental morphology in both location and shape.

To further analyze the driving mechanisms behind these fracture paths, the total damage accumulated at the critical integration point is decomposed into its two constituent parts, as defined by the extended GTN framework (Equation (9)). Throughout the simulation, the competing weight function $\beta \left(\eta ,\;\bar{\theta }\right)$ dynamically partitions the total damage increment between the void-dominated mechanism and the shear-dominated mechanism. The term void damage, denoted $D_{V}$, refers to the accumulated contribution from the classical GTN void growth and nucleation terms, ${\mathrm{df}}_{\mathrm{gr}}+{\mathrm{df}}_{\mathrm{nucl}}$, weighted by $\beta (\eta ,\;\bar{\theta }\;)$. Physically, $D_{V}$ represents the damage arising from volumetric void expansion driven by hydrostatic tension and from strain-controlled particle debonding. The term shear damage, denoted $D_{s}$, refers to the accumulated contribution from the N-H shear term ${\mathrm{df}}_{\mathrm{shear}}$, weighted by $\left[1-\beta (\eta ,\;\bar{\theta }\;)\right]$, and represents the damage caused by void distortion and elongation within localized shear bands. The sum $D_{total}=D_{V}+D_{s}$ corresponds to the total void volume fraction f that is tracked by the GTN yield function and ultimately determines the onset of macroscopic fracture when $f\geq f_{f}$.

**Figure 16:**
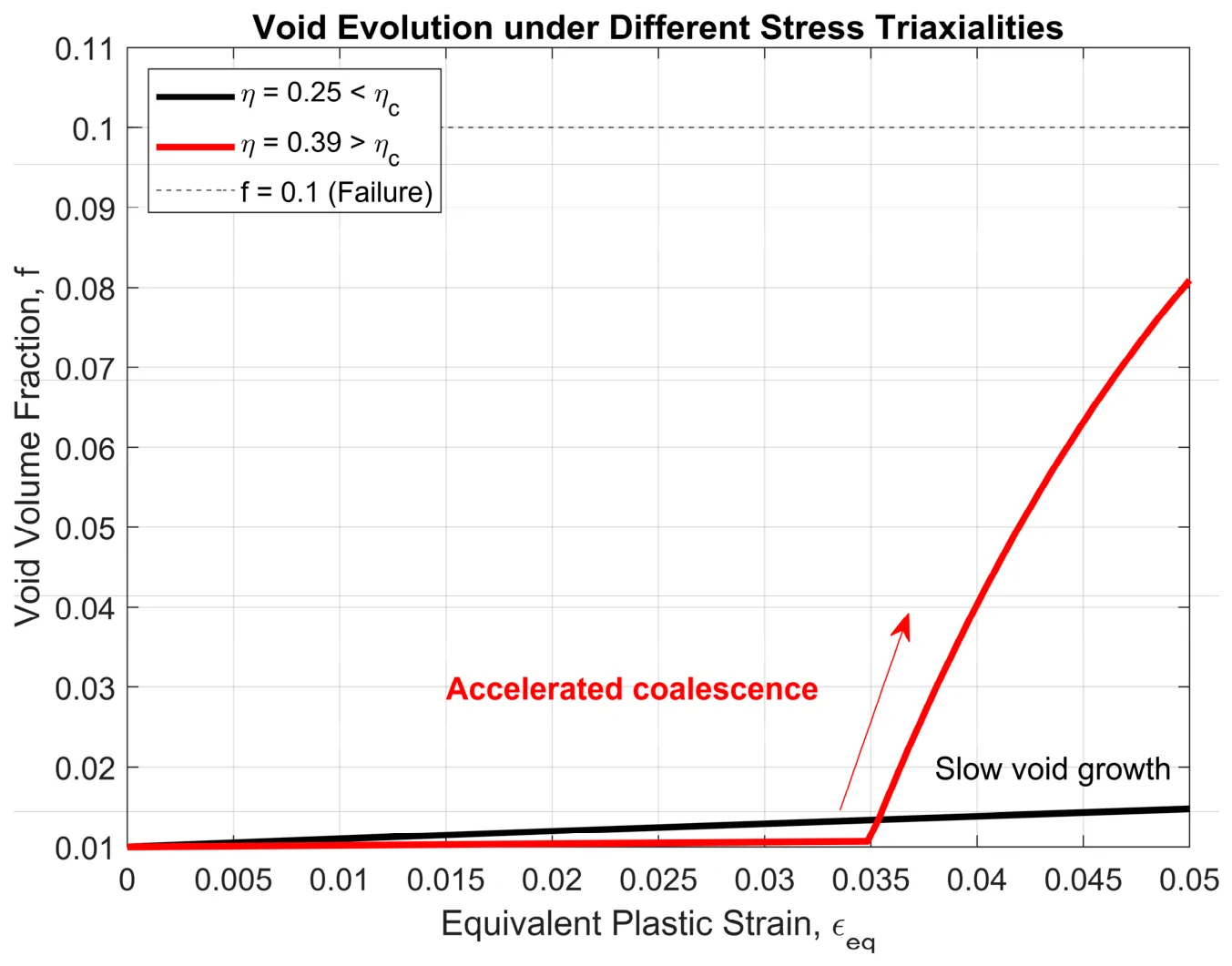
Evolution of the void damage component as a function of equivalent plastic strain governed by the competing weight function.

**Figure 17:**
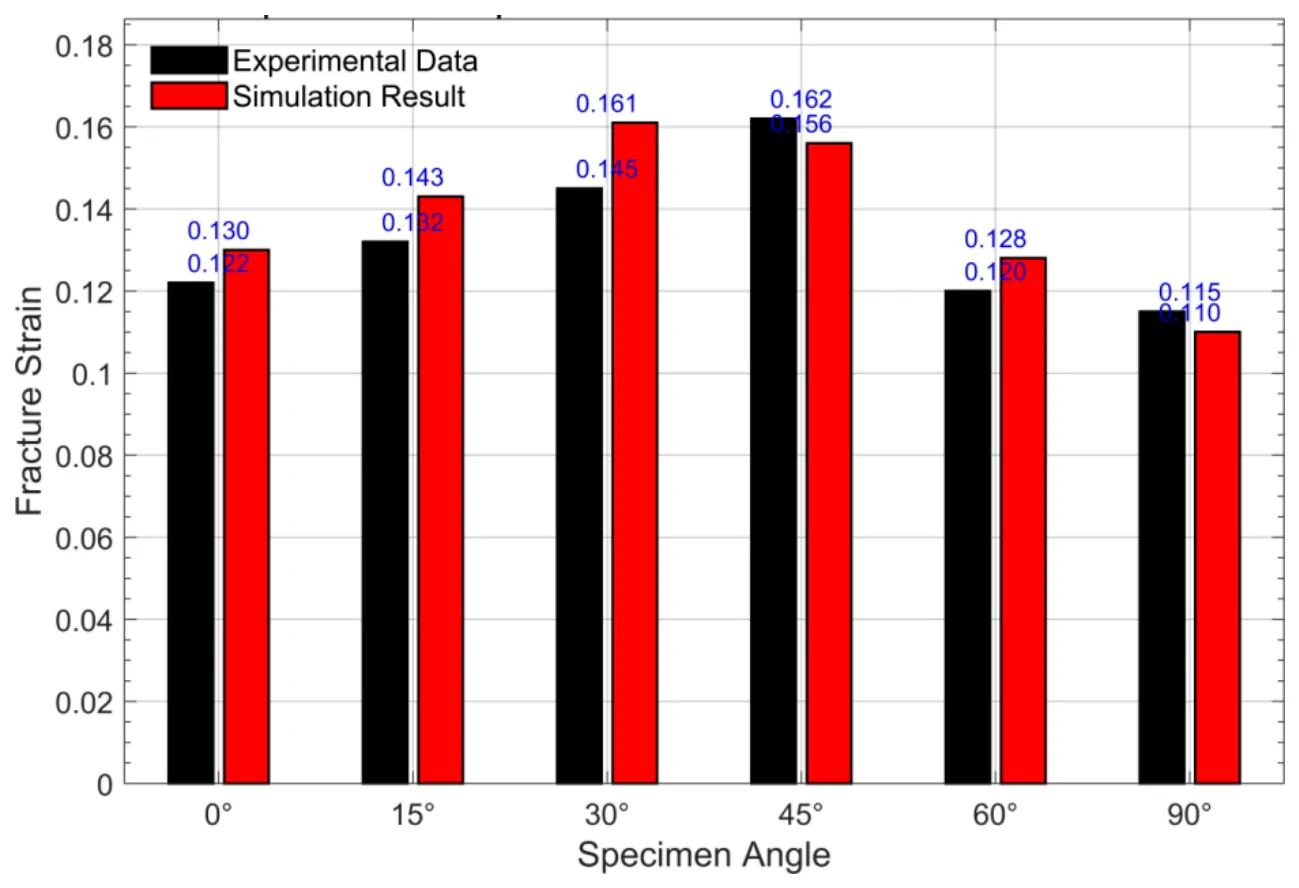
Validation of predicted fracture strains against experimental measurements.

Damage evolution remains moderate under low triaxiality, driven primarily by shear mechanisms. Once η exceeds this threshold, the void damage weight increases rapidly, accelerating accumulation and softening until failure—a nonlinear trend reflecting the physical response of the DP1180 microstructure. The evolution of the void damage component with equivalent plastic strain is shown in Figure 16. Finally, Figure 17 compares predicted and experimental fracture strains against average stress triaxiality. The model shows good agreement with data across the entire stress state range, particularly capturing the non-monotonic transition around η ≈ 0.35, which validates the efficacy of the competitive weight function in describing mixed-mode fracture.

#### 5.2.3. Effect of the Normalized Lode Angle Parameter

**Figure 18:**
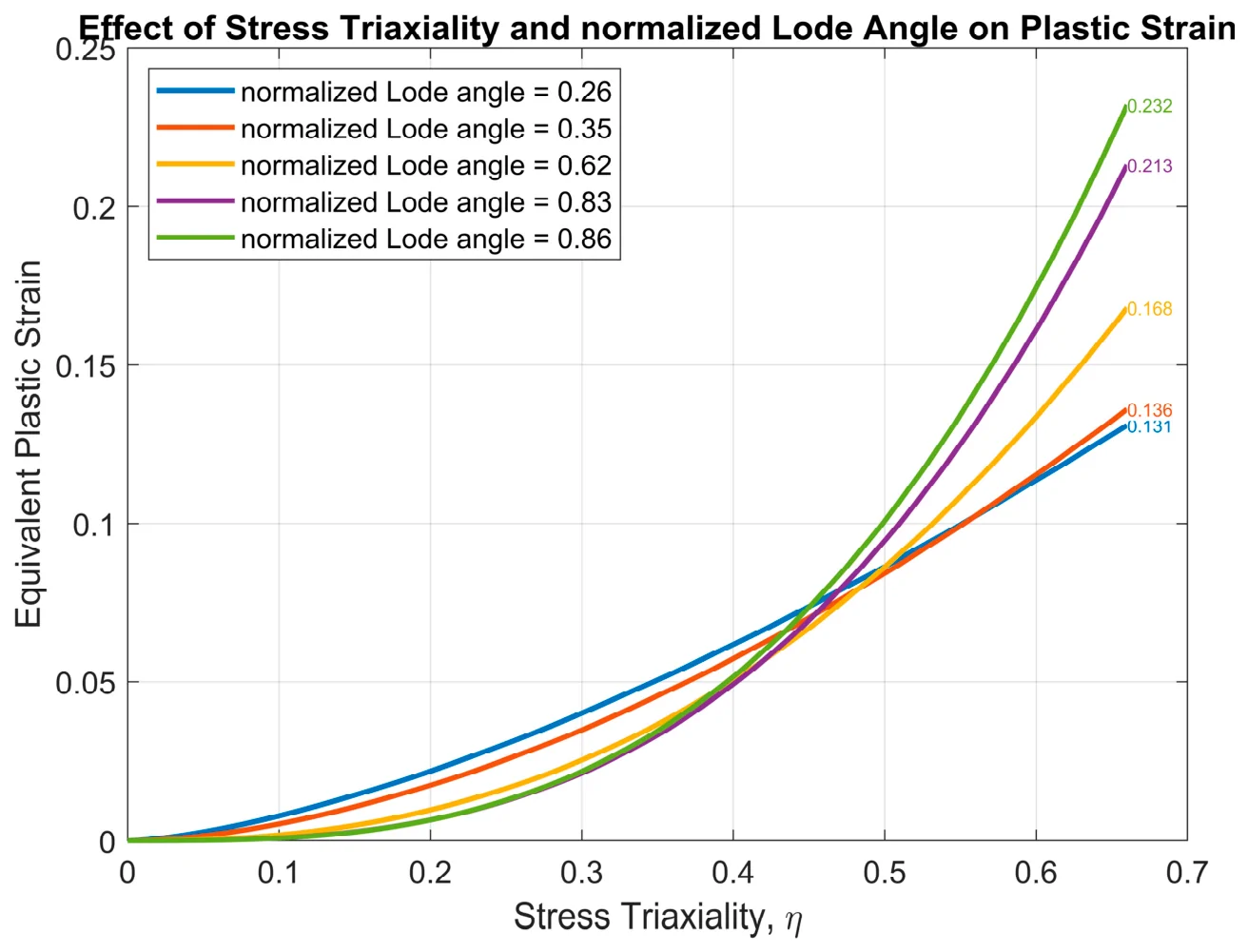
Evolution of ductile fracture strain with varying normalized Lode angle.

The influence of the normalized Lode angle on equivalent plastic strain was quantified via numerical simulations of Nakazima tests. As illustrated in Figure 18, fracture strain exhibits distinct stress-state dependence across varying normalized Lode angles. In the low triaxiality regime, the curves converge, suggesting negligible Lode sensitivity under low hydrostatic pressure. Conversely, at higher triaxialities, significant divergence is observed; for instance, at identical high triaxiality, equivalent plastic strain drops from 0.232 to 0.131 as the normalized Lode angle decreases, corresponding to a 43% reduction. Consistent with the proposed competitive mechanism, lower normalized Lode angles amplify shear damage weight, precipitating earlier failure, whereas higher angles favor stable void growth and enhanced ductility. This trend reflects the underlying physical mechanisms governing complex stress paths in DP1180 steel.

### 5.3. Tension-Bending Experiments

**Figure 19:**
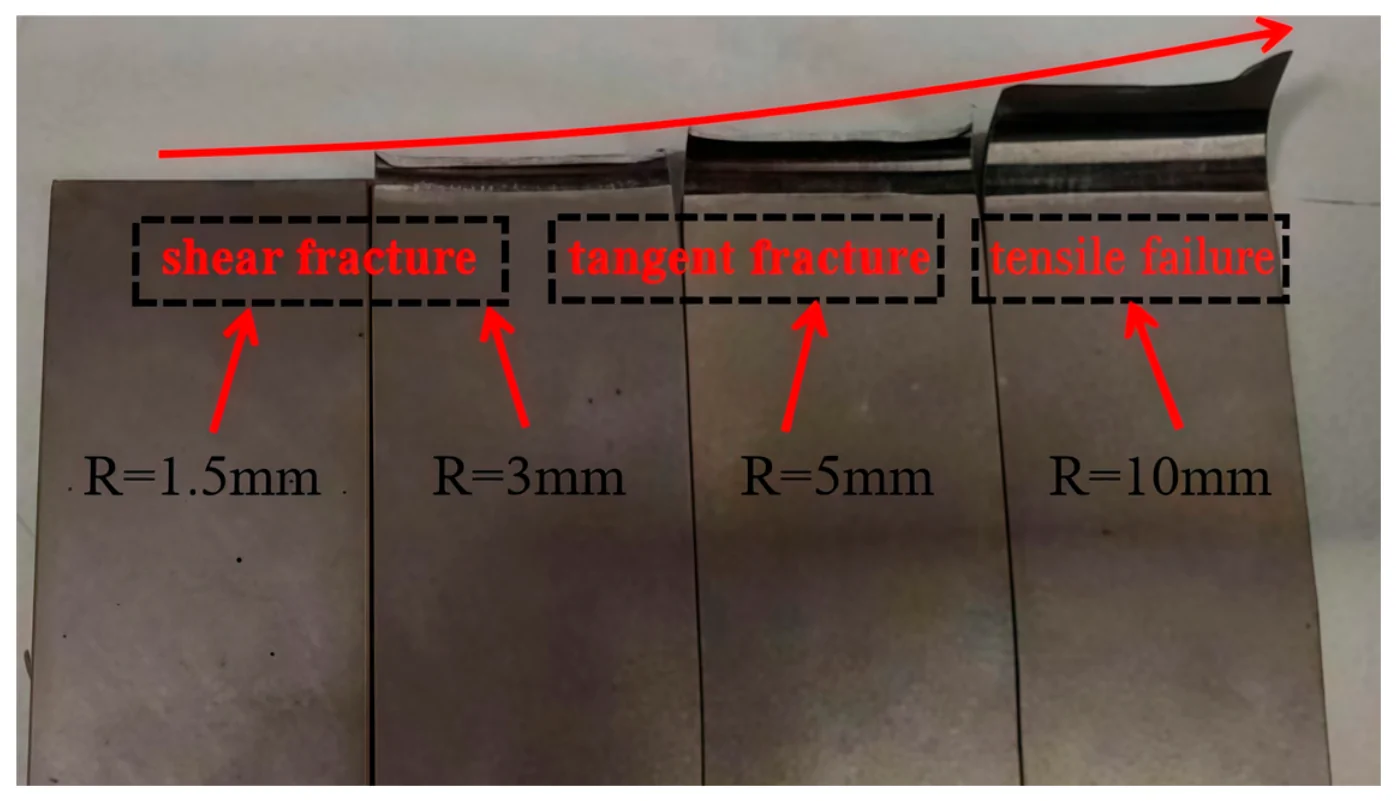
Fracture mode transition driven by stress state evolution via varying punch radii in tension-bending.

Combined tension-bending tests were performed on 1.0 mm thick rectangular specimens using a hydraulic press to simulate stamping conditions and broaden the stress state space. A blank holder force of 10 kN balanced wrinkle suppression with material flow. As illustrated in Figure 19, varying the punch corner radius induced three distinct fracture modes in the fillet region. Small radii (1.5 and 3 mm) promoted intense shear deformation, resulting in smooth shear fracture, whereas a large radius (10 mm) approximated uniaxial tension, leading to ductile tearing with significant necking. An intermediate radius of 5 mm produced a mixed-mode fracture, combining features of both shear and tensile mechanisms.

### 5.4. Finite Element Analysis of Tension-Bending

**Figure 20:**
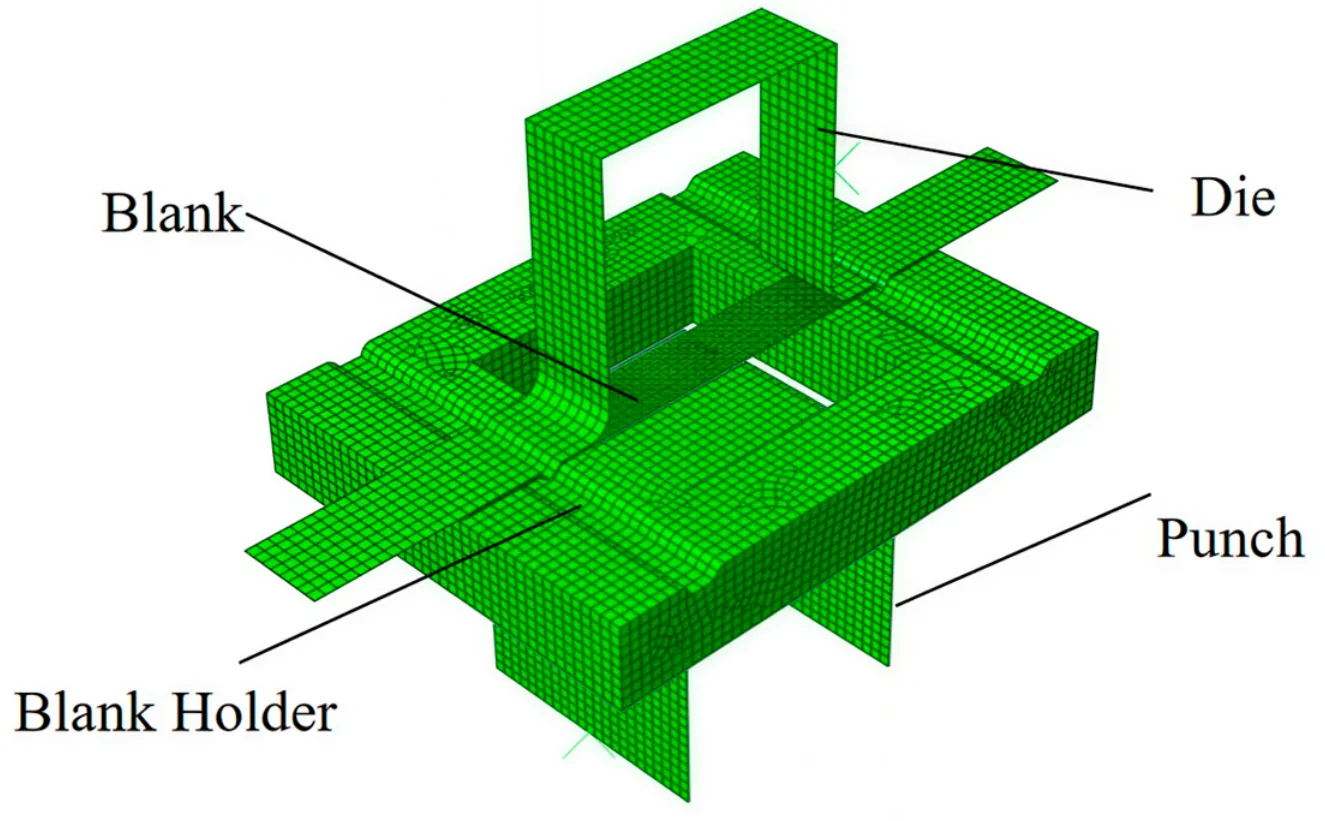
Finite element discretization for the tension-bending specimen.

A VUMAT subroutine incorporating the Hill’48-GTN-coupled damage model was implemented via a stress update compensation algorithm. The finite element model, illustrated in Figure 20, employed C3D8R reduced-integration hexahedral elements, with mesh refinement to 0.8 mm in the central region transitioning to 2 mm peripherally to balance accuracy and efficiency. Displacement-controlled loading was applied to the mid-section using calibrated parameters to simulate fracture propagation.

**Figure 21:**
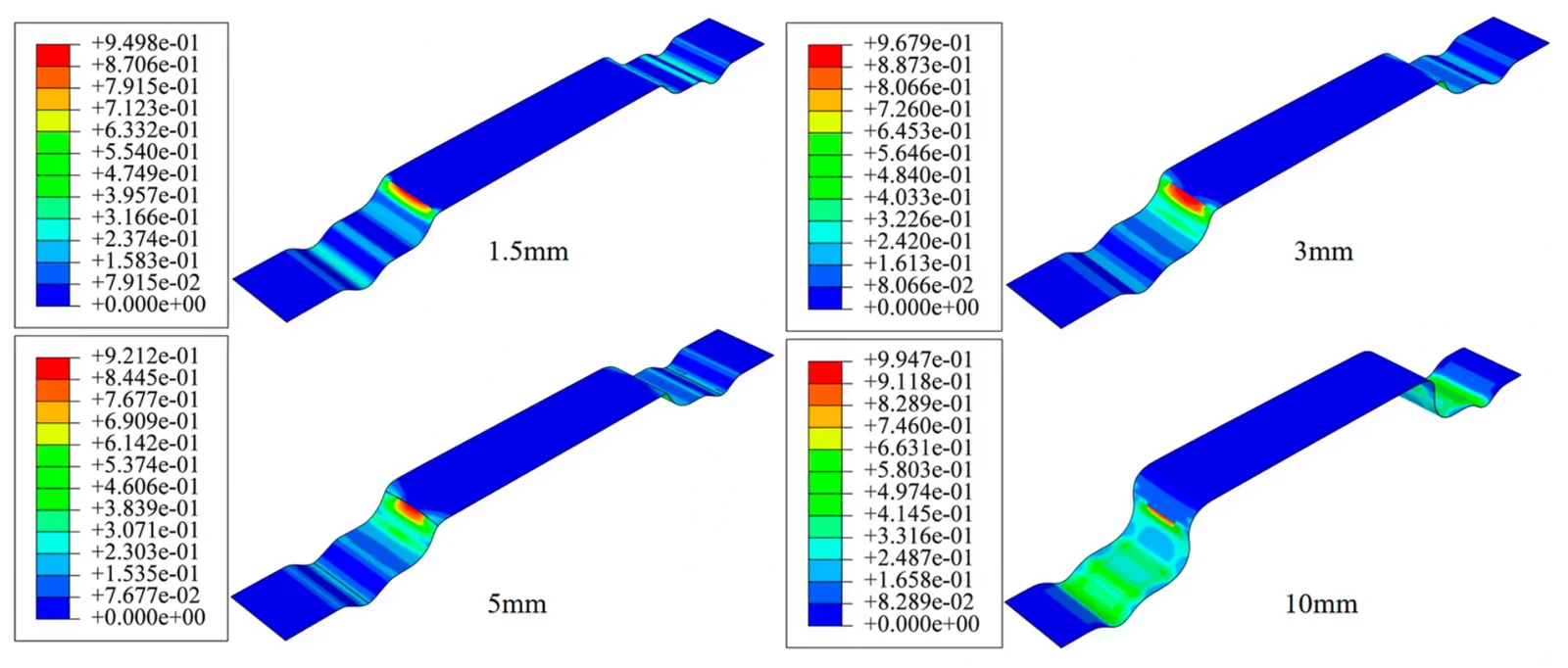
Evolution of crack trajectories in tension-bending specimens.

Damage distributions at fracture, shown in Figure 21, evolve systematically with increasing punch radius. Small radii induced localized damage in narrow bands with straight crack paths, indicative of shear fracture. An intermediate radius of 5 mm initiated damage at the fillet-side wall tangent, propagating along an inclined path with slight tip blunting, suggesting mixed-mode behavior. In contrast, a large radius of 10 mm concentrated damage at the sheet center with significant necking, driving a crack perpendicular to the tensile direction typical of ductile tearing, as the punch radius increases, the fracture mode transitions from shear-dominated to tension-dominated failure.

**Figure 22:**
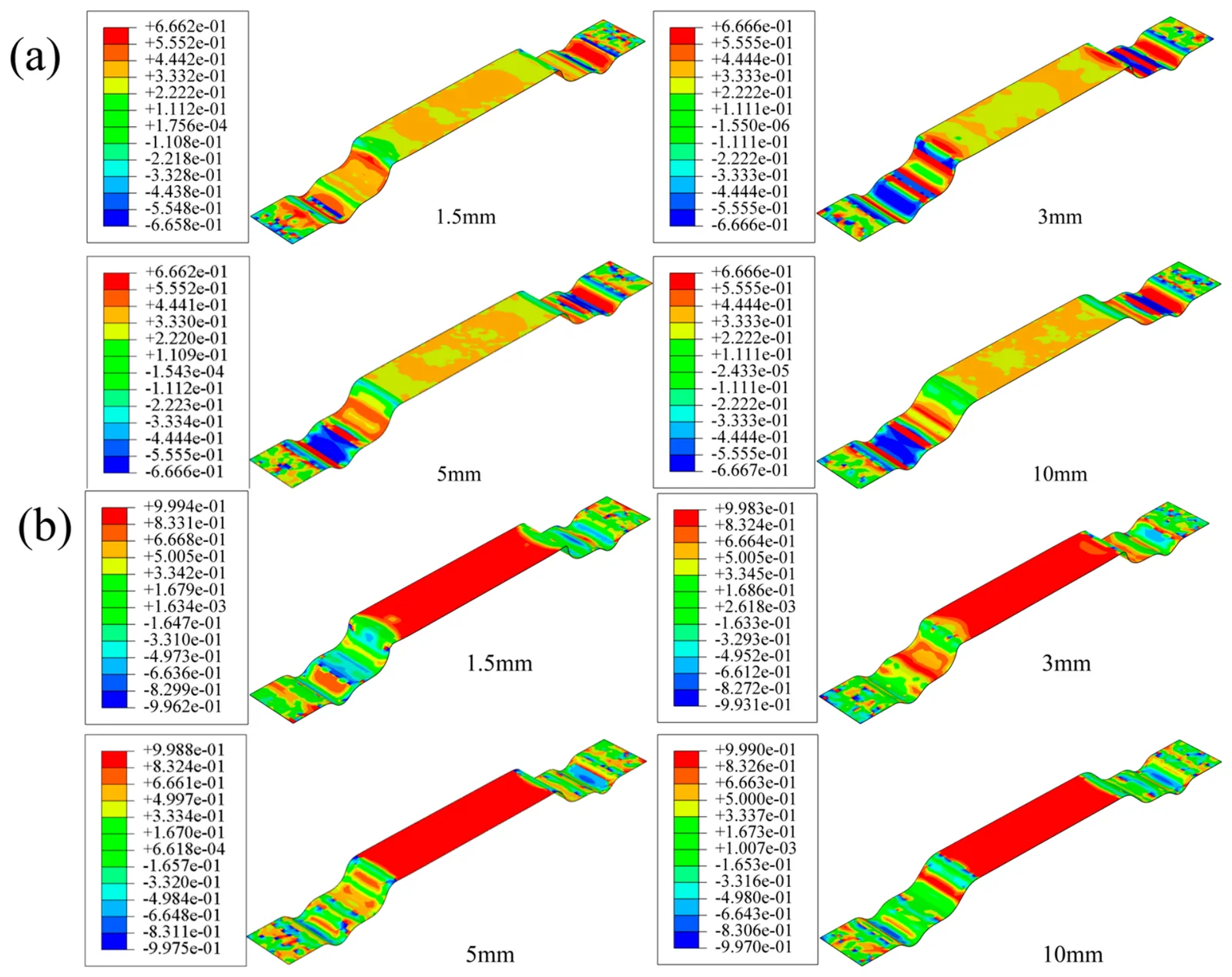
Stress triaxiality (**a**) and normalized Lode angle (**b**) distributions under varying punch radii.

To correlate these modes with local stress states, average stress triaxiality and normalized Lode angle at fracture were extracted from key integration points and mapped onto a fracture strain diagram derived from standard notched and Nakazima specimens. As presented in Figure 22, tension-bending data occupy distinct regions: small radii correspond to low triaxiality and high normalized Lode angle, aligning with shear-dominated low fracture strains; the large radius falls within high triaxiality and low Lode angle, characterizing void-coalescence-driven ductile fracture, while the intermediate case resides near the critical triaxiality of 0.35 with Lode angle between 0.4 and 0.6, locating it within the transition zone. These results suggest that geometric constraints shift local stress states to drive fracture mode transitions. By coupling triaxiality and Lode angle effects on both void and shear damage, the proposed model effectively reproduces the continuous transition from shear fracture to ductile tearing.

## 6. Conclusions

An extended GTN framework integrating Hill’48 anisotropy and the Nahshon–Hutchinson shear mechanism was developed to describe competitive shear-tension fracture in AHSS under complex stamping conditions. A novel weight function, governed by stress triaxiality and the normalized Lode angle, was formulated to dynamically allocate damage contributions. Experimental results indicate a transition from shear-dominated to void-dominated fracture as triaxiality increases, with an intermediate mixed-mode regime centered around a critical threshold of η ≈ 0.35. This threshold is characterized by inflection points in both fracture strain and micro-morphology, marking the onset of void mechanism predominance. The proposed physically constrained function ensures smooth transitions between failure modes. Validation against hierarchical calibration data demonstrates that the model reproduces stress–strain responses and fracture paths for both standard and complex tension-bending specimens. These findings suggest that the approach effectively describes fracture mode transitions under evolving stress states, offering a practical framework for engineering the analysis of AHSS-forming processes. The current validation is limited to DP1180 sheets at room temperature under quasi-static loading; application to other AHSS grades, thicknesses, strain rates, or temperatures requires recalibration.

## Figures and Tables

**Table 1:** Chemical composition of DP1180 steel (wt.%).

C	Si	Mn	P	S	Al
0.12	0.16	2.21	0.014	0.001	0.03

**Table 2:** Calibrated anisotropic and hardening parameters for DP1180 steel.

F	G	H	L	M	N	K	n
0.55	0.46	0.46	1.5	1.5	1.5	1543	0.07

**Table 3:** Identified material constants for the extended GTN model.

$a_{1}$	$a_{2}$	$\eta_{c}$	$q_{1}$	$q_{2}$	$q_{3}$	$\varepsilon_{N}$	$S_{N}$	$k_{\omega }$	$f_{n}$	$f_{c}$	$f_{f}$
8.5	−1.5	0.35	1.5	1	2.25	0.3	0.1	1.5	0.018	0.023	0.1

## Data Availability

The data presented in this study are available on request from the corresponding author due to restrictions related to industrial collaboration agreements and the proprietary nature of the alloy compositions investigated.
